# Differential crosstalk between toxin-immunity protein homologs divides *Myxococcus* nonself siblings into close and distant social relatives

**DOI:** 10.1128/mbio.03902-24

**Published:** 2025-03-28

**Authors:** Feng Wang, Jing Luo, Zheng Zhang, Ya Liu, Duo hong Sheng, Li Zhuo, Yue-zhong Li

**Affiliations:** 1 State Key Laboratory of Microbial Technology, Institute of Microbial Technology, Shandong University, Qingdao, China; 2 Suzhou Research Institute, Shandong University, Suzhou, China; Northern Arizona University, Flagstaff, Arizona, USA

**Keywords:** toxin–immunity protein system, multiple homologous pairs, cross-immunity, kin discrimination, close and distant relationship, *Myxococcus xanthus*

## Abstract

Many bacteria discriminate self and nonself using toxins and their corresponding immunity proteins. The toxin–immunity systems often include homologs, potentially creating crosstalk with unknown influences on kin discrimination. In this study, we investigated the kinship controlled by four homologous toxin–immunity systems in the social bacterium *Myxococcus xanthus*. We determined that the four homologous systems each play an independent role in the discrimination of self and nonself. However, the immunity proteins inactivate not only the corresponding nuclease toxin proteins but also some non-corresponding toxin proteins, depending on their sequence and structural similarities. The nonself relatives controlled by toxin–immunity proteins with or without crosstalk exhibit differential co-growth and collaborative behaviors. We concluded that differential crosstalk between toxin–immunity protein homologs can divide bacterial nonself lineages into close and distant relatives displaying differential collaboration and antagonistic behaviors.

**IMPORTANCE:**

This study significantly contributes to our knowledge of kin selection and social behavior in bacteria. The interactions between four homologous toxin–immunity protein systems of *Myxococcus xanthus* were investigated, and evidence was obtained that these systems can distinguish between self and nonself cells within a species. Importantly, this study revealed that nonself lineages, which display varying degrees of genetic relatedness, can co-grow and collaborate in distinct patterns. This discovery implies that the differential crosstalk between homologous toxin–immunity proteins can mimic the degree of kinship; through this activity, bacteria can differentiate close and distant relatives. This novel insight into bacterial social dynamics and kin discrimination supports kin selection theory and enriches our knowledge on microbial interactions and evolutionary strategies. These findings have broad implications for microbial ecology, evolution, and the development of cooperation strategies.

## INTRODUCTION

Bacteria living in the environment must compete for space and essential nutrients and have developed an array of offense-and-defense mechanisms (1), including different kinds of toxins and their corresponding immunity protein systems (2–4) to bolster their competitive advantages. Antimicrobial toxins are secreted by bacterial cells through associated secretion systems (1, 5–7), such as the type VI secretion system (T6SS), a nanomachine equipped in a broad range of gram-negative bacteria (8–10). The toxin-producing cells encode immunity proteins respectively corresponding to toxin proteins as a self-protection strategy to prevent intoxication of their own or neighboring cells (6, 11). These toxin–immunity protein systems mediate interbacterial wars and function as a mechanistic basis for bacterial cell-to-cell discrimination of self and nonself within species (10, 12–16). Many bacterial species have evolved multiple toxin–immunity protein homologs in generally a similar process (5), which potentially creates crosstalk between homologs with unknown influences on kin discrimination.

*Myxococcus xanthus* is a gram-negative gliding predatory bacterium with complex multicellular population behavior. The strains of this bacterium typically live in social groups in soil, where the clonal cells cooperatively move and feed on prey microbes, and when nutrients are depleted, they undergo a developmental program that involves fruiting body morphogenesis and myxospore formation (17, 18). Cooperating with self-entities while excluding incompatible nonself material is imperative for *M. xanthus* to maintain social integrity; for this purpose, the bacterium has developed multiple kin discrimination strategies, including the T6SS, the outer membrane exchange, and undefined rearrangement hotspot systems (12, 19–21). In our previous studies, we inserted the pMiniHimar-lacZ transposon randomly into the *M. xanthus* DK1622 genome to screen for deficiency in kin discrimination via colony boundary formation and revealed eleven deficient mutants from 3,392 mutations (22). The kin discrimination mutants with an insertion at a locus can form colony boundaries with not only the wild-type strain but also the mutant strains that have the insertion at other loci (22). Among the eleven mutants, eight were inserted by the transposon at six homologous genetic loci. We demonstrated that the deficiency in kin discrimination is associated with nuclease toxin proteins and the corresponding immunity proteins in two mutants (SI01 and SI08), where the toxins are delivered via the T6SS with the help of a PAAR protein (12) or N-terminal PAAR domain (19).

In this study, we investigated how kinship is controlled by four homologous toxin–immunity protein pairs. We determined that these four systems are independently involved in the discrimination of self and nonself in *M. xanthus*. We demonstrated that the immunity proteins can not only inactivate the nuclease and cytotoxic activities of the corresponding toxin proteins but also cross-neutralize some non-corresponding toxins. The relatives controlled by toxin–immunity protein pairs with crosstalk can co-grow and co-develop fruiting bodies in mixed cultures, whereas the relatives with no or weak crosstalk of their toxin–immunity protein pairs can hardly co-grow and/or co-develop fruiting bodies. Our results highlight that bacteria can employ differential crosstalk between homologous toxin–immunity proteins to distinguish nonself lineages into close and distant relatives with different behaviors in collaboration and antagonism.

## RESULTS

### Independent roles of four homologous toxin–immunity systems in kin discrimination

The six homologous genetic loci encode toxin and immunity proteins, each conjugated with a *PAAR* gene, except for the locus inserted in the SI02 mutant (Fig. S1). The PAAR protein is a carrier for the secretion of toxin effectors through the T6SS (23). The *PAAR* gene identified in the SI08 mutant of *M. xanthus* DK1622 encodes a PAAR protein with a C-terminal extended domain (19), whereas the other four loci encode single-domain PAAR proteins, including the locus in the SI01 mutant (12). We selected the four toxin–immunity protein pairs that are conjugated with a single-domain *PAAR* gene to investigate their potential crosstalk and influence on kin discrimination. We use the terms Toxin1–4 and corresponding Imm1–4 for the four toxin and immunity proteins, respectively, and the studied toxin protein (MXAN_0050) and immunity protein (MXAN_0049) in the SI01 locus were termed Toxin1 and Imm1, respectively.

To determine the roles of the toxin and immunity proteins in kin discrimination, we deleted their genes in the *M. xanthus* DK1622 genome. In theory, inactivation of the immunity proteins should lead to cell death because of the cytotoxicity of toxin proteins. However, the transposon insertions in the kin discrimination mutants of *M. xanthus* revealed in our previous screening (22) were generally located at the predicted immunity genes that are located downstream of the toxin genes (refer to Fig. S1). Further deletion of the *MXAN_0049* (*Imm1*) (12) or *MXAN_RS24590* (19) immunity gene similarly produced viable kin discrimination mutants. Notably, similar phenomena have also been observed in other bacteria, such as *Proteus mirabilis* (24) and *Pseudomonas aeruginosa* (25, 26), but the mechanisms involved are still unclear. Consistently, deleting each of the other three of the four immunity genes from the DK1622 genome similarly resulted in the desired viable mutants. We determined by sequencing that, compared with the gene sequences deposited in the NCBI database, i.e. *Toxin1* (Gene ID: 41357559), *Toxin2* (Gene ID: 41358754), *Toxin3* (Gene ID: 41364307), and *Toxin4* (Gene ID: 41359504), the sequences of the toxin genes are unchanged in the mutants. In addition, these toxin genes are similarly transcribed after the deletion of the immunity genes (Data set S1). The viability of the deletion mutants of immunity genes suggests some unknown compensatory pathways in prevention of intoxication, requiring further investigation.

**Fig 1:**
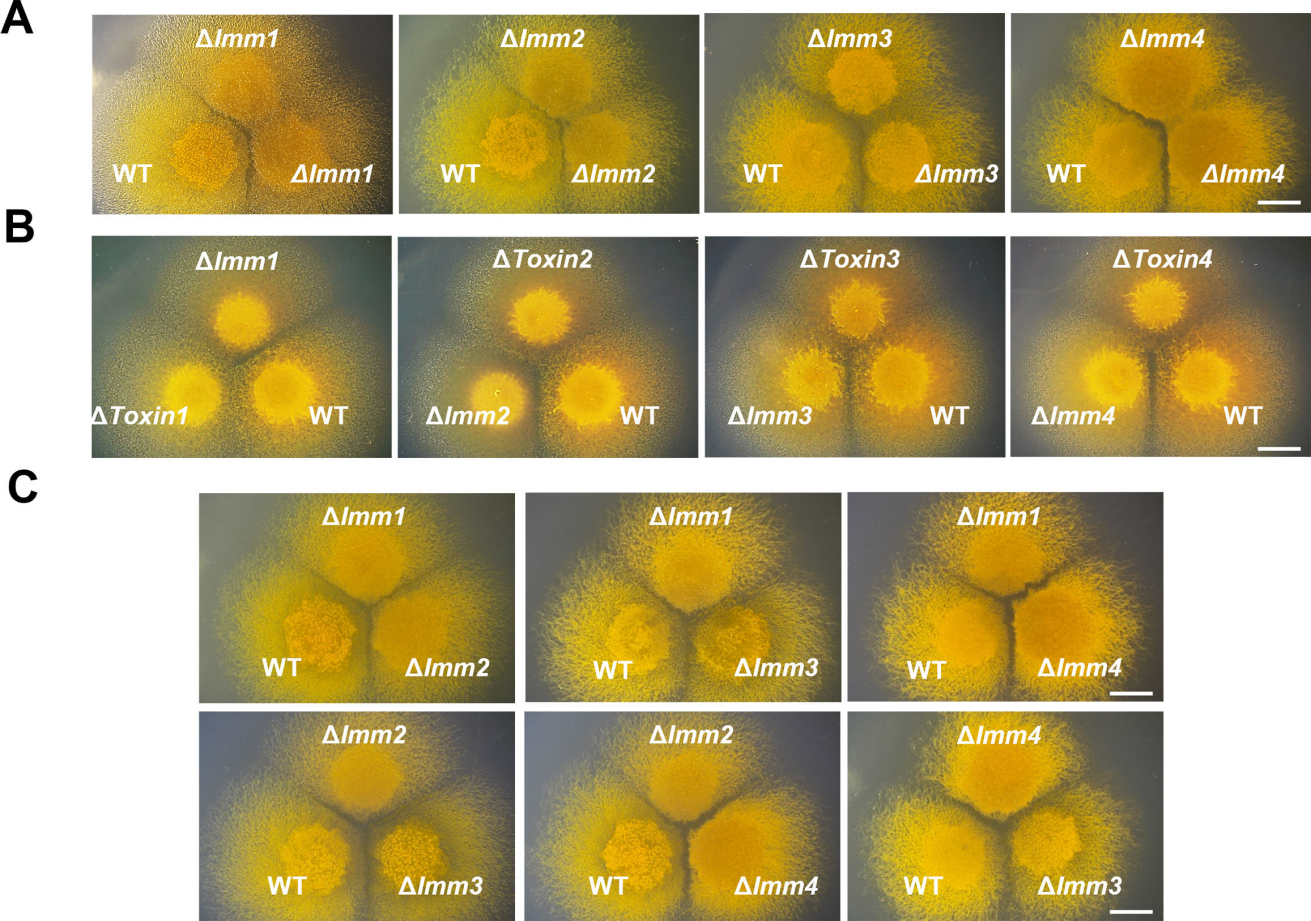
Four homologous toxin‒immunity protein pairs mediate *Myxococcus* kin discrimination. The culture images show colony boundary formation between the immunity protein-encoding gene deletion strains and the wild-type strain (A), immunity protein-encoding gene deletion strains and the corresponding toxin gene deletion strains (B), and different immunity protein-encoding gene deletion strains (C). The scale bar represents 5 mm.

The formation of colony boundaries, termed colony merger incompatibility (27), is an important ecological phenotype for bacterial cells to distinguish self from nonself. On CTT growth media, the Δ*Imm1–4* mutants all formed visible colony boundaries when paired with the wild-type strain (Fig. 1A), displaying phenotypes almost identical to those observed in the corresponding transposon–insertion mutants previously reported (22). Deletion of the toxin-encoding genes in DK1622 caused the mutants to merge colonies with not only the corresponding immunity protein mutants but also the wild-type strain DK1622 (Fig. 1B). These results suggested that the immunity genes and their paired toxin genes are involved in kin discrimination in *M. xanthus*. When the *Imm1–4* mutants were inoculated pairwise in proximity, they all formed incompatible colonies with each other (Fig. 1C), suggesting that the four immunity genes are each a determinant playing an independent role in kin discrimination.

In many bacteria, the *PAAR* gene is located at a genetic locus adjacent to the T6SS components (8). However, in *M. xanthus* DK1622, the *PAAR* genes are all conjugated with toxin–immunity protein genes and are separated from the single T6SS locus (*MXAN_4800-MXAN_4813*). We knocked out the four *PAAR* genes in DK1622, respectively, resulting in Δ*PAAR1–4* mutants, termed according to the nomenclature of conjugated *Toxin–Imm1–4*. As expected, the colonies of these *PAAR* deletion strains merged with those of the wild-type and the mutants deficient in the adjoint but not the allopatric immunity genes (Fig. S2). These results were similar to those of the mutants with the deletion of cognate toxin-encoding genes (Fig. 1B), indicating the exclusive loading role of the four *PAAR* genes for the secretion of associated toxins, thereby independently involved in kin discrimination.

### The toxic and paired immune characteristics of the four toxin–immunity systems

**Fig 2:**
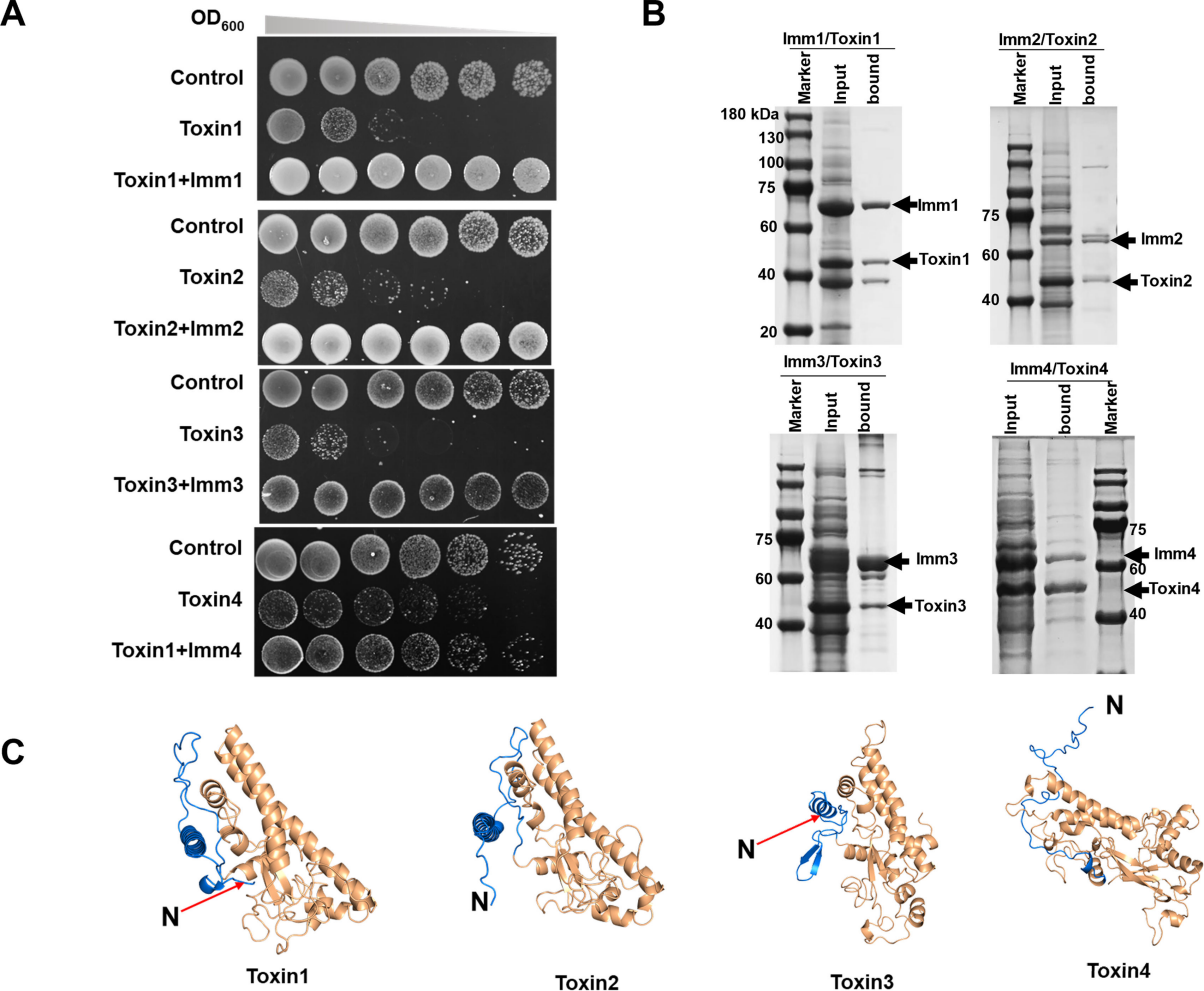
Functional interactions between the toxin protein and its corresponding immunity protein. (A) Expression of a toxin gene alone or together with its corresponding immunity protein-encoding gene in *E. coli*. The protein expression was controlled by an IPTG-inducible promoter. The starting culture had an OD of 0.1, and the samples were diluted twofold in the gradient. (B) Interaction analysis between immunity proteins and their corresponding toxins. The immunity protein (but not the toxin protein) was designed to contain an MBP tag. Input mixtures (the supernatant from the cell lysate) containing both toxin and immunity proteins were eluted through the amylose resin. The amylose resin-bound and amylose resin-unbound samples were analyzed via SDS-PAGE, followed by Coomassie blue staining. Control groups expressing only toxin or immunity proteins were also tested (Fig. S3). (C) Model structures of the four toxin proteins, as predicted by AlphaFold2, showing highly diverse N-terminal regions, marked in blue.

The above phenotypic results indicate that the four homologous toxin–immunity protein systems play independent roles in the ability to distinguish self and nonself in *M. xanthus*. To determine the *in vivo* toxic and immune characteristics, we heterologously expressed the toxin and corresponding immunity proteins in *Escherichia coli*. The toxin genes are each under the control of an IPTG-induced promoter. Upon the addition of IPTG, the growth of *E. coli* strains harboring toxin genes was markedly inhibited by the induction of toxin gene expression, and this growth inhibition was alleviated when the corresponding immunity genes were co-expressed (Fig. 2A).

To assess the binding ability between toxin and immunity proteins in cells, we co-expressed their genes in *E. coli* cells using two plasmids, one encoding a SUMO-tagged toxin protein and the other encoding the corresponding immunity protein tagged with MBP. The supernatant from the cell lysate was eluted through the amylose resin. (Fig. S3 shows the expression of SUMO-tagged toxin proteins and MBP-tagged immunity proteins. Because toxin proteins are free of MBP, their retention on the amylose resin depends on their ability to bind to MBP-containing immunity proteins. As shown by SDS-PAGE (Fig. 2B), the toxin proteins remained on the resin together with their corresponding immunity proteins.

Toxin1 (MXAN_0050) is an AHH nuclease (12, 23). Docking modeling revealed that the four toxin proteins have highly similar binding patterns to dsDNA molecules (Fig. S4), suggesting that the other three toxins (Toxin2–4) are also nucleases. To obtain proteins for *in vitro* assays, we attempted to overexpress and purify toxin and immunity proteins in *E. coli*. However, while the immunity proteins were soluble and were easily purified, the toxin proteins were largely insoluble when expressed alone.

We found that while the main bodies of the four toxin proteins are highly similar, the N-terminal regions (40–70 amino acids) are highly varied in the sequence and structure (Fig. 2C; detailed sequence alignment is shown in Fig. S5). If the N-terminal fragments are removed, the truncated toxin proteins, termed Toxin1–4^D^, have a similar dsDNA-binding pattern to that of the full-length toxin proteins (Fig. S4). Similarly, protein complex modeling also revealed that deletion of the N-terminal regions had small effects on the formation of toxin–immunity protein complexes (Fig. S6). We deleted only the N-terminal region of the four toxin genes in *M. xanthus* DK1622, and these mutant strains exhibited similar colony boundary phenotypes as the mutants in which the whole toxin gene was deleted (Fig. S7). These results suggested that the non-conserved N-terminal fragments of the toxin proteins probably play a special role in the secretion of the conjugated toxin proteins.

We fused an MBP tag at the N-terminus of the truncated toxin proteins and overexpressed them in *E. coli* BL21, which allowed us to purify the truncated toxin proteins (Fig. S8). The *in vitro* experiment revealed that the truncated toxin proteins could completely degrade the DK1622 genomic DNA substrates within 40 minutes at 37°C (Fig. S9), which is similar to the results obtained with the whole Toxin1 (MXAN_0050) protein (12).

### Differential crosstalk between the homologous toxin–immunity proteins

**Fig 3:**
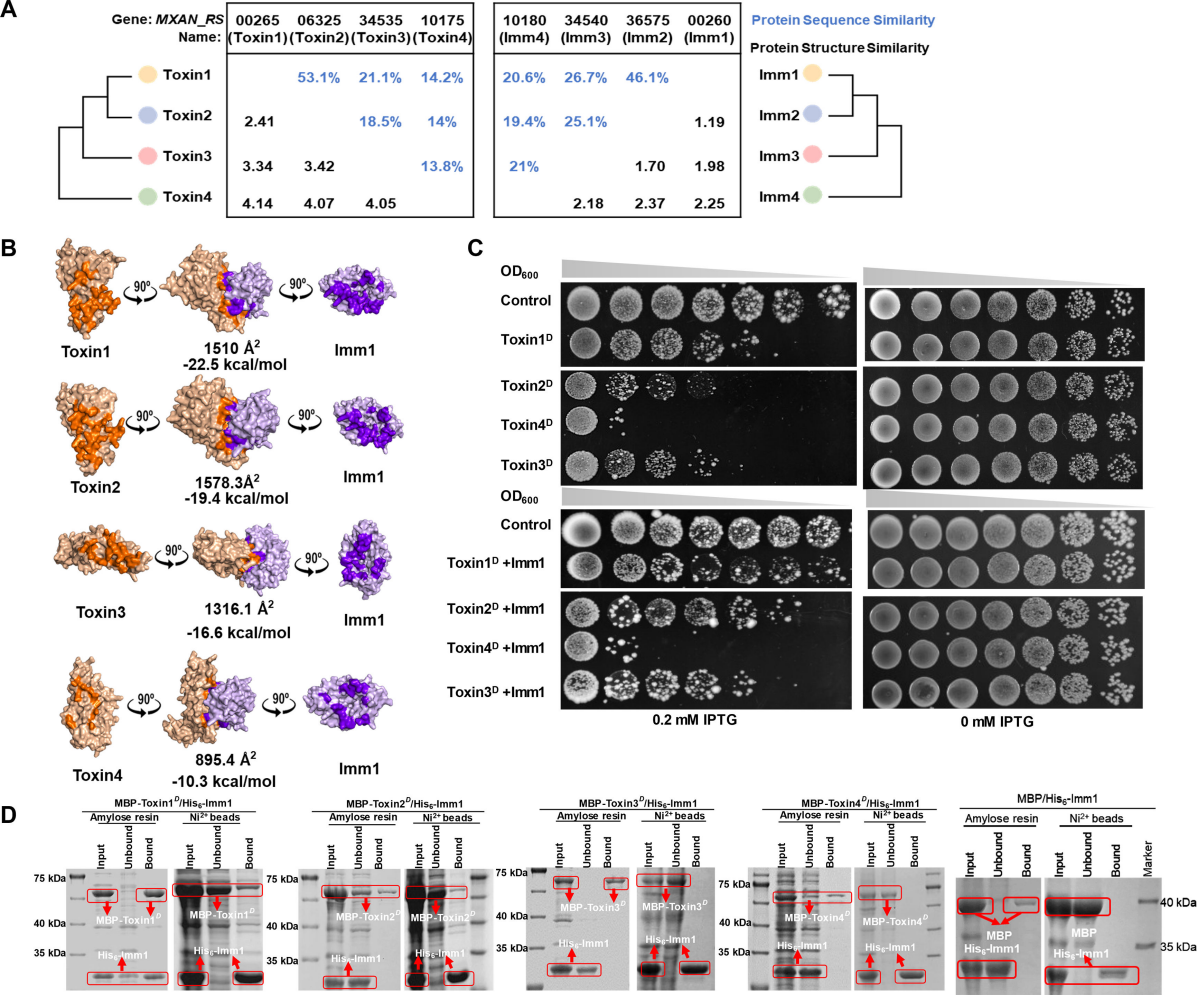
Cross-interaction between toxin–immunity protein pairs. (A) Diagram illustrating the evolutionary relationships of the four toxin and immunity proteins. The figures in blue represent protein sequence similarity, and the figures in black indicate structural RMSD (root-mean-square deviation). An RMSD less than 2.0 Å typically suggests structural similarity between two proteins, and a value greater than 4.0 Å suggests noticeable structural differences. (B) Modeling the complex formation of Imm1 with different toxin proteins. The toxin protein is shown in brown, and Imm1 is shown in purple. The amino acid sites involved in the interaction are highlighted in bright brown for the toxin protein and dark purple for the immunity protein. The values below the complex represent the interaction surface area (in Å^2^) and the protein binding free energy (in kcal/mol). (C) Cross-reactivity between Imm1 and different truncated toxin proteins (Toxin1D–4D) co-expressed in *E. coli*. Toxin protein expression was induced by the addition of IPTG. The control group contained only the plasmid without toxin genes. “0.2 mM IPTG” represents induced protein expression. The starting culture had an OD of 0.2, followed by a fivefold dilution for inoculation. (D) Cross-interaction between different toxin proteins fused with an MBP tag and the His_6_–Imm1 protein. The pulldown assay was based on the binding of either MBP to the amylose resin or His_6_ to Ni²^+^ beads. A control experiment was used to test the binding of MBP-toxin to Ni beads as a reference (Fig. S10).

The four toxins are all alkaline, and the immunity proteins are acidic (Table S1). We found that the sequence and structure of these four homologous toxin and immunity protein pairs correspondingly changed, generally in a decreasing order from Toxin1–Imm1 to Toxin4–Imm4 (Fig. 3A). For example, the sequence similarity is 53.1% between Toxins 1 and 2, 21.1% between Toxins 1 and 3, and 14.2% between Toxins 1 and 4. Concordantly, the sequence similarity is 46.1% between Imms 1 and 2, 26.7% between Imms 1 and 3, and 20.6% between Imms 1 and 4 (a detailed sequence comparison of the toxins and immunity proteins is provided in Fig. S5A and B). The coevolution of the paired toxin and immunity proteins not only suggests that they share a common ancestor, thereby preserving the immunity capacities against toxins, but also suggests potential crosstalk between homologous pairs.

We modeled the complexes between the immunity protein Imm1 and each of Toxins 1–4 to estimate their binding ability (Fig. 3B). Generally, a larger interaction surface and greater binding free energy indicate a stronger and more stable complex. We found that, although the interaction interface of Imm1 with Toxin1 was slightly smaller than that of Imm1–Toxin2 (1510 Å^2^ vs 1578.3 Å^2^), the binding free energy was greater (−22.5 kcal/mol vs −19.4 kcal/mol). Comparatively, the interaction interface and the binding free energy were both much lower for the protein complex of Imm1–Toxin3 (1316.1 Å^2^ and −16.6 kcal/mol) and lowest for Imm1–Toxin4 (895.4 Å^2^ and −10.3 kcal/mol). The gradient variation suggests that the binding strength decreases roughly according to the sequence and structural variations of the toxin–immunity protein pairs.

We tested the *in vivo* cross-immunity of Imm1 against different toxin proteins in *E. coli* (Fig. 3C). For the experiments, two plasmids were constructed into *E. coli* cells. The expression of the toxin gene, controlled by an IPTG-induced promoter, was in the pMAL c5X plasmid, while the Imm1 gene was ligated into the pET28a plasmid under the T7 promoter control. The control strains similarly contained the two plasmids, without the Imm1 gene in the pET28a plasmid or without the toxin and Imm1 genes in both plasmids. When 0.2 mM IPTG was added to induce toxin gene expression, the growth of *E. coli* strains harboring toxin genes was strongly inhibited. Interestingly, compared with the growth inhibition of *E. coli* cells caused by the expression of toxin genes alone, the coexpression of Imm1 could alleviate the growth inhibition caused by Toxin1 and Toxin2, had almost no effect on Toxin3, and showed no effect at all on Toxin4. These findings demonstrated that, in addition to the highly specific interaction between toxin and its corresponding immunity protein, cross-immunity may occur between an immunity protein and some non-corresponding toxins.

We further performed pull-down experiments using His_6_-Imm1 and four MBP-tagged truncated toxin proteins (Fig. 3D; MBP/His_6_–Imm1 was used as a control). The truncated toxin proteins all bound to the amylose resin via the MBP tag, whereas the His_6_–Imm1 protein bound to the Ni^2+^ beads by the His_6_ tag. We found that Toxin1^D^ and Toxin2^D^ both appeared in the Imm1-bound fraction on the Ni^2+^ beads, whereas the Toxin3^D^ and Toxin4^D^ proteins remained in the unbound fraction. However, on the amylose resin, the Imm1 proteins appeared only in the Toxin1^D^-bound fraction. When the MBP-tagged toxins were incubated with Ni^2+^ beads alone, they barely bound to the beads (Fig. S10).

Notably, although the complex modeling predicted that each of the four toxin proteins had a binding ability to the Imm1 protein, the coexpression of Imm1 could not alleviate the growth inhibition by Toxin3 or Toxin4 in *E. coli*, and the pull-down assay could not capture the Imm1-binding complex with the Toxin3^D^, Toxin4^D^, or even Toxin2^D^ proteins. These results probably reflect the sensitivity of different methods. Nevertheless, the above results indicate that crosstalk may occur between homologous kin discrimination systems, which depends on their sequence and structural similarities, i.e.*,* interaction abilities. In addition, we also noticed that the crosstalk similarly influenced the colony boundary phenotypes; some were vague, such as the boundary between Δ*Imm1* and Δ*Imm2*, whereas some were distinct, such as the boundary between Δ*Imm1* and Δ*Imm4*. According to Hamilton’s rule, benefits are given more to close than to distant relatives (28, 29). The crosstalk, as well as the phenotypic variation in the colony boundary, suggests differential social behaviors of antagonism or collaboration between the nonself relatives of *M. xanthus*.

### Differential co-growth and co-development abilities between kin discrimination mutants

To assess potential differences in the social behavior of strains in mixtures, we introduced a kanamycin (Km) resistance gene into *M. xanthus* mutants that are deficient in one of the four immunity genes, named Δ*Imm1–4^Km^*. The use of antibiotic resistance markers allowed us to assess the impact of mixed growth on individual strains. These kanamycin-resistant strains were pairwise mixed in equal amounts with the kanamycin-sensitive strains of the mutants deficient in one of the four immunity genes, named Δ*Imm1–4*. The mixtures were incubated on CTT agar media for 2 days to test the growth competition between paired strains. When mixed with the same Δ*Imm* strains, the four Δ*Imm^Km^* strains showed no significant difference in growth (*t* test, *P* > 0.05), as determined by counting colony formation units (CFUs) on kanamycin selection media (Fig. S11). Similarly, the growth of the Δ*Imm1–4^Km^* strains was slightly affected when the strains were mixed with their corresponding toxin gene mutants. However, when mixed with the wild-type strain DK1622, the CFUs of kanamycin-resistant mutants were greatly decreased (Fig. S11). These results indicated that, owing to the loss of immunity protein genes, the *Imm* mutants were less fit in terms of competition with the wild-type strain, which is similar to the results for the Δ*Toxin1* (*MXAN_0050*) and Δ*Imm1* (*MXAN_0049*) mutants (12).

**Fig 4:**
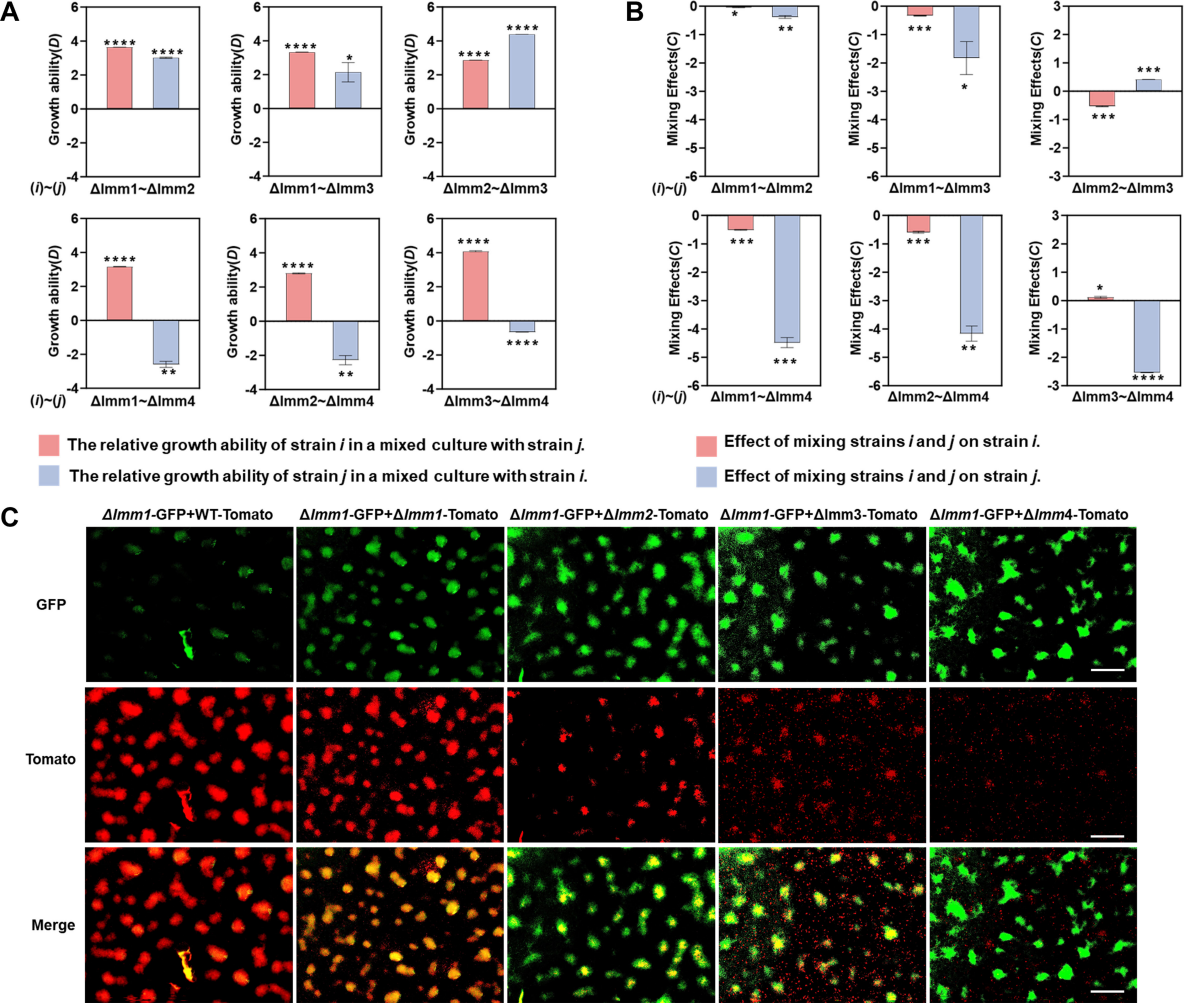
Co-growth and co-development behavior between immunity protein mutants. (A) Relative growth ability (*Dij*) in pairwise mixtures of immunity protein mutants. A positive D value indicates that the strain can coexist with the other strain in the mixture, whereas a negative D value suggests that the strain cannot coexist with the other strain. (B) Relative mixing effect (C*ij*) in pairwise mixtures of immunity protein mutants. A positive C value indicates that a strain is promoted by the other strain in the mixture, whereas a negative C value indicates that a strain is inhibited by the other strain. The error bars in panels A and B represent the standard deviation, and the asterisks denote *P* values for *t* tests of differences from 0: *P < 0.05, **P < 0.01. ***P  <  0.001; ****P  <  0.0001. (C) Fruiting body development in pairwise mixtures of the GFP-marked Δ*Imm1* strain and the tomato-marked wild-type and Δ*Imm1–4* strains. The mixtures were incubated on TPM agar plates for 7 days. The scale bar represents 120 µm.

Interestingly, when mixed with different *Imm* mutant strains, the Δ*Imm1–4^Km^* strains presented variable coexisting and interacting abilities. We used *Di*[*j*] and *Ci*[*j*] values (22, 30) to measure the relative growth abilities of individual strains in a mixture: a positive or negative *Di*[*j*] value indicates whether strain *i* can or cannot coexist with strain *j* in coculture, whereas a positive or negative *Ci*(*j*) value indicates that the growth of strain *i* is promoted or inhibited by strain *j*. In two-strain cocultures of Δ*Imm1*, Δ*Imm2,* and Δ*Imm3*, the *Di*[*j*] values were positive for both partners after 2 days of incubation, suggesting their ability to coexist. However, although the existence of Δ*Imm4* had a positive *Di*[*j*] value for Δ*Imm1*, Δ*Imm2,* or Δ*Imm3*, the presence of Δ*Imm1*, Δ*Imm2,* or Δ*Imm3* was negative for the coexistence of Δ*Imm4* (Fig. 4A). This indicates that Δ*Imm1*, Δ*Imm2*, and Δ*Imm3* can coexist in pairwise combinations, whereas Δ*Imm4* is unable to coexist with any of them. Although Δ*Imm1*, Δ*Imm2,* and Δ*Imm3* are capable of coexistence, the mixing effects (*Ci*[*j*]) showed that the growth of a strain is mostly inhibited by the presence of a competitor, except for weak positive effects of the presence of Δ*Imm2* on Δ*Imm3* and Δ*Imm4* on Δ*Imm3* (Fig. 4B). In the pairwise mixture of Δ*Imm4* and Δ*Imm1–3*, the presence of Δ*Imm1–3* had a strong negative effect on the growth of Δ*Imm4*, but the presence of Δ*Imm4* generally had a weak effect on the growth of Δ*Imm1–3*.

We further marked the Δ*Imm1* strain with the GFP gene and the wild-type and the four immunity protein mutants with the tomato gene to track kin discrimination-deficient mutants during fruiting body development (Fig. 4C). In the mixture of Δ*Imm1*-GFP and WT-tomato, almost only the WT cells were observed in the fruiting body structure after 7 days of incubation on TPM. In comparison, the Δ*Imm1*-GFP and Δ*Imm1*-tomato cells both presented a fruiting body structure in their mixture after incubation. Similarly, both Δ*Imm1*-GFP and Δ*Imm2*-tomato cells were detectable in the same fruiting bodies, indicating that these two nonself relatives codeveloped the structure. In the mixture of Δ*Imm1*-GFP and Δ*Imm3*-tomato, the Δ*Imm3* cells were also involved in the formation of fruiting bodies together with Δ*Imm1*-GFP. However, many free Δ*Imm3*-tomato cells were present outside the Δ*Imm1*–Δ*Imm3* fruiting body structures. When Δ*Imm1*-GFP was mixed with *Imm4*-tomato, fruiting bodies developed only from the Δ*Imm1*-GFP cells, whereas the remaining *Imm4*-tomato cells were outside of the fruiting body structure. In summary, Δ*Imm1* can develop mixed fruiting bodies with Δ*Imm2* and Δ*Imm3* to different extents but never with Δ*Imm4*; this is consistent with the abovementioned co-growth results. Taken together, differential crosstalk between toxin–immunity protein homologs distinguishes the nonself lineages of *M. xanthus* into close and distant social relatives with differential collaboration and antagonistic behaviors.

## DISCUSSION

Bacterial cells live in close proximity within microbial communities, where many bacterial processes occur in the public sphere, such as the secretion of enzymes for the degradation of complex nutrients and the release of toxins or antibiotics for killing or repelling ambient competitors (31, 32). Compared with multicellular organisms, kin identification and selection might be more important for bacterial cells that live in the environment. To occupy the ecological niche and share public goods, bacteria have developed diverse antibacterial toxins and corresponding immunity proteins for kin discrimination, as well as interbacterial antagonism (2, 5, 11). In bacteria, kin discrimination is the process by which bacterial cells distinguish ambient individual cells as self or nonself; this process leads to discrepant behaviors, such as coexistence and cooperation within self and repelling or killing behaviors against nonself (31, 33–36). Bacterial relatedness is generally identified by a genetic locus encoding a toxin and its corresponding immunity proteins, and the loss-of-function mutation of the immunity gene leads to unrecognition (10, 12–16). Seemingly, kinship in bacteria is limited to simple self and nonself discrimination. However, from a large number of pairwise examinations of diverse *Bacillus* species against *B. subtilis*, Lyons and Kolter suggested that *B. subtilis* can distinguish degrees of nonkin relatedness (37). Our data reported here indicate that differential crosstalk between toxin–immunity protein homologs can be responsible for distinguishing nonself lineages into close and distant relatives.

In bacterial genomes, genes encoding toxin and immunity proteins generally occur pairwise in the same gene cluster, suggesting the importance of immunity proteins for neutralizing toxin toxicity in bacterial cells (2, 11). The four studied toxin–immunity protein systems are homologous in terms of their sequence and structure and play independent roles in the discrimination of self and nonself in *M. xanthus*. We found that immunity proteins can inactivate not only the corresponding toxin proteins but also some noncorresponding toxins according to their sequence and structural similarities. Stronger interactions were observed when the sequence and structural differences of the homologs are smaller. The strength of the interaction between immunity proteins and toxins, in turn, influences their ability to antagonize toxin toxicity and thereby enables cells to survive together. Crosstalk can weaken the strength of competition between nonself lineages, thus dividing nonself relatives according to different kinships. The relatives controlled by toxin–immunity proteins with crosstalk can co-grow and co-develop fruiting bodies in mixed cultures, whereas the relatives with no or weak crosstalk cannot. The potential crosstalk between multiple toxin and immunity protein homologs might provide some compensatory pathways to prevent intoxication. More importantly, the crosstalk between toxin–immunity homologs can differentiate bacterial nonself lineages into close and distant relatives, thus performing different behaviors in collaboration and antagonism.

Hamilton’s kin selection theory falls into two summary terms: fitness and genetic relatedness, i.e., the degree of shared alleles is a measurement of population discrimination and social behavior (28, 29, 38). The degree of kinship (relatedness) is *r* between relatives; the donor provides a benefit (*b*) to the recipient at a cost (*c*) to itself by one another’s fitness, requiring *br* > *c*. Hamilton’s rule and the associated concept of inclusive fitness have been extremely successful in explaining and modeling social evolution, including the evolution of social behavior and social discrimination. Differential crosstalk between toxin–immunity homologs divides bacterial relatives with a hierarchical relationship. The close and distant kinships between bacterial nonself relatives provide more complex and delicate balances between cooperation and competition and may confer evolutionary advantages by enabling certain lineages to coexist and establish stable cooperative associations under environmental conditions. Crosstalk between homologous recognition systems can be especially beneficial in diverse ecological niches, where selectively excluding nonself competitors while collaborating with closely related strains enhances the resource efficiency and resistance against external challenges. The generation of relative recognition specificity and hierarchy can help bacteria, such as myxobacteria, construct sophisticated social communities.

Myxobacteria are predominantly distributed in soil and water sediments worldwide (39–41). Myxobacteria normally possess large genomes and often encode the largest number of toxin–immunity protein systems (8, 42–44). We comparatively analyzed the homologs of the gene clusters of *Toxin–Imm1–4* in completely sequenced *Myxococcus* genomes. These gene clusters are widely distributed across the *Myxococcus*, particularly in *M. xanthus* and other closely related species, but with different numbers and composition of the gene cluster (Data set S2). In addition, we previously complemented the Δ*MXAN_0049* mutant with a homologous gene from *M. fulvus* HW-1 (*LILAB_08510*, 86% identity with *MXAN_0049*), and the complemented strain merged colonies with DK1622 but formed a boundary with the Δ*MXAN_0049* mutant (12). These results suggested that toxin–immunity homologs might function similarly in different *Myxococcus* strains. The multiple toxin–immunity protein pairs encoded in myxobacterial genomes are thought to establish a self-identity barcode for individual strains (21). These self-identity barcodes (21) provide many possibilities for distinguishing self and nonself as well as the close and distant relationships of nonself lineages. We propose that the crosstalk between homologous toxin–immunity protein pairs should be attributed to the sequence and structural similarities of these homologs; stronger interactions are observed when the sequence and structural differences are smaller. Differential crosstalk among these toxin–immunity protein homologs divides nonself siblings into close and distant relatives in terms of social relationships. Close relatives can coexist in mixed cultures and develop mixed fruiting bodies, while distant relatives cannot; this activity may result in synergistic coevolution among within-group lineages (45) and nonself lineages of close relatives.

## MATERIALS AND METHODS

### Bacterial strains and culture conditions

The *Myxococcus* strains and mutants were cultivated at 30°C in liquid CTT media (10 g/L casein peptone, 1.97 g/L MgSO_4_7H_2_O, 1 mM PBS buffer, and 10 mM Tris HCl buffer [pH 7.6]) with shaking at 200 rpm or on CTT plates supplemented with 1.5% agar (46). The *E. coli* strains in this study included BL21(DE3) for protein expression and DH5α for the conservation of other plasmids.

The *E. coli* strains were cultivated in liquid Luria–Bertani (LB) medium with shaking at 200 rpm or on solid LB medium plates at 37°C (47). When needed, final concentrations of kanamycin (Km; 40 µg/mL), ampicillin (Amp; 100 µg/mL), or chloramphenicol (Cm; 34 µg/mL) were added to the CTT and LB media. The plasmid information for the strains used in the experiment is included in the supplementary materials.

### Construction of the *M. xanthus* mutants

In-frame deletion of the gene was performed in *M. xanthus* DK1622 via positive–negative KG cassettes (48). The cassettes consisted of a Km resistance gene for positive screening and a galactokinase gene (*galK*) for negative screening to replace the genomic target gene by double-crossover homologous recombination. Genomic DNA from DK1622 served as the template for PCR amplification of the upstream and downstream regions of the target gene. The upstream and downstream regions were fused to create internal deletion fragments, which were subsequently cloned and inserted into the plasmid pBJ113 via overlap PCR. The deletion plasmids were electroporated into DK1622, in which they were integrated into the genome by single homologous recombination, and colonies grown on CTT agar plates containing Km were selected. These strains were then cultured on 1% D-Gal CTT agar plates in the absence of Km, and the unstable tandem duplication excised the plasmid via homologous recombination. The screened strains retained either the original wild-type locus or the deleted locus, depending on where the recombination occurred. The obtained strains were further screened via colony PCR and sequencing (12). The Δ*Imm1^Km^* mutant, the Δ*Imm2^Km^* mutant, the Δ*Imm3^Km^* mutant, and the Δ*Imm4^Km^* mutant were generated via electroporation of the plasmid pSWU19 (containing the Km resistance gene), which can integrate into the Mx8 *attR* site of the DK1622 genome. The *attR* primer pair was used for screening (49). The primer information used for constructing the mutant strains is provided in Table S2. Plasmid construction was performed using the ClonExpress II One Step Cloning Kit (Vazyme), which employs a homologous recombination-based method to enable efficient and seamless assembly of DNA fragments for generating the desired plasmids and mutants.

### Mixing experiments for vegetative growth

The cells were shaken in liquid CTT growth medium at 30°C for 24 hours until they reached the mid-log phase. After centrifugation, the harvested cells were resuspended in TPM buffer and adjusted to a density of 5 × 10^9^ cells/mL. The cell suspensions of the paired strains were mixed at a 1:1 (vol/vol) ratio. Five-microliter aliquots of the mixed suspensions were dropped onto CTT agar media. After 48 hours of incubation at 30°C, the entire colonies were harvested, suspended in 500 µL of TPM buffer, and then 10-fold serially diluted in TPM buffer. To calculate the number of surviving partner cells, the cell diluents (50 µL) were mixed with 3 mL of molten CTT soft agar and poured onto CTT hard agar plates with Km or with no added antibiotic. After 3–5 days of incubation at 30°C, the number of CFUs during vegetative growth was counted. Three dilutions and three technical replications for each dilution were used for counting. The statistical analysis of the competitive ability between incompatible strains was adapted from previously described methods (30), with minor modifications.

The number of mutant cells is denoted by *N*. In the single-culture condition, it is denoted *Ni*, whereas in the mixed-culture condition, it is *Nij*. The initial cell count before mixing was labeled (*t_0_*), and the cell count after 2 days of coculture was labeled (*t_2_*). Thus, the growth capacity (*D*) of the mutant cultured alone was calculated as follows:


(1) $$Di=log\left[Ni\left(t_{2}\right)/Ni\left(t_{0}\right)\right]$$

The growth capacity (*D*) of the mutant strain after coculture was as follows:


(2) $$Di\left(j\right)=log\left[Nij\left(t_{2}\right)/Nij\left(t_{0}\right)\right]$$

The positive value of *Di*(*j*) indicates that strain *i* can grow and survive in the presence of strain *j*, whereas a negative value indicates that strain *i* cannot grow and survive with strain *j*.

To compare the relative performance of the two strains within each competing pair, we calculated *Ci*(*j*) (relative mixing effect). The difference in the mixed-growth capability *Ci(j*) between the *i* mutant strain and the *j* strain during cocultivation is as follows:


(3) Cij=Di(j)−Di

A positive value of *Ci*(*j*) indicates that strain *i* promotes growth in the presence of strain *j*, whereas a negative value indicates that strain *i* is inhibited by strain *j*. Graphs and statistical analyses were performed in GraphPad Prism 9.0.2.

### Strain co-development

The GFP/tomato gene was introduced into wild-type WT and immunity protein mutant cells, which were subsequently cultured in CTT liquid media at 200 rpm and 30°C for approximately 20 hours. Pairwise combinations of strains were mixed in equal proportions and cocultured on TPM agar for approximately 7 days until fruiting bodies formed. The color of the fruiting bodies was observed via fluorescence microscopy.

### Boundary assays

The strains were cultured in liquid CTT media with shaking at 200 rpm. After 20–24 hours, the cells were harvested by centrifugation and suspended in TPM buffer at a final concentration of 5 × 10^9^ cells/mL. Then, 3 µL aliquots of the cells were inoculated on CTT agar plates at an intercolony distance of 7 mm. After 3–5 days of cultivation, the boundaries were observed under an SMZ100 dissection microscope.

### Protein expression and purification

The constructed expression vector was transformed into *E. coli* BL21 (DE3) and spread onto antibiotic plates. A single clone of the transformant was picked and cultured in the LB liquid medium at 37°C with shaking at 200 rpm overnight. The following day, 2% LB culture was inoculated into fresh LB liquid medium (with the required antibiotic), and the mixture was cultured at 37°C with shaking at 200 rpm until the OD_600_ reached approximately 0.6–0.8. IPTG (0.2 mM) was added to induce expression. After an additional 20 hours of incubation at 16°C, the cells were collected via centrifugation at 8,000 rpm for 10 minutes and then washed with the resuspension buffer (20 mM Tris-HCl [pH 7.4], 200 mM NaCl and 5% glycerol). The cells were resuspended in resuspension buffer and lysed via sonication on an ice slurry (30% amplitude, 5 seconds on/10 seconds off, total 10 minutes). The mixture was centrifuged at 12,000 rpm for 30 minutes to remove intact cells and debris. For the His_6_-tagged protein, the supernatant was incubated with Ni^2+^ beads (GE Healthcare, Sweden) for 1 hour at 4°C, and then the beads were washed with resuspension buffer supplemented with 50 mM imidazole. The bound proteins were eluted with a 100–250 mM imidazole gradient in the resuspension buffer. For the MBP-tagged protein, the supernatant was incubated with the amylose resin (New England Biolabs) for 2 hours at 4°C, and then the resulting mixture was washed with resuspension buffer. The fusion protein was eluted with resuspension buffer supplemented with 10 mM maltose. If necessary, the purified proteins were combined and concentrated via a 10 kDa centrifugal concentrator (Millipore, Germany). The protein concentration was measured using a NanoDrop One spectrophotometer (Thermo Fisher) at 280 nm with a path length of 1 mm.

### Toxicity assay in *E. coli*

A toxicity assay was performed as described previously (50). The toxin genes were expressed under the control of an IPTG-inducible promoter in the pMAL c5X plasmid. For the co-expression of the toxin and the corresponding immunity proteins, the conjugated two genes were constructed into the pMAL c5X plasmid under the IPTG-inducible promoter control. The empty pMAL c5X plasmid was transformed into the BL21 strain as a control. Notably, in the cross-immunity experiments, we introduced two plasmids into an *E. coli* cell: one toxin gene was ligated into the pMAL c5X plasmid, while the Imm1 gene was ligated into the pET28a plasmid under the control of the T7 promoter. As a control, the empty plasmids pMAL c5X and pET28a were transformed into *E. coli*. In the strains expressing only a toxin, the empty plasmid pET28a was also included.

The *E. coli* strains were cultured overnight at 37°C in LB liquid media supplemented with Amp. The OD_600_ values of all the strains were measured, and the strains were subsequently adjusted to the same cell density in fresh LB medium. All strains were serially diluted. Next, 5 µL of the cell dilutions was spotted on LB agar plates containing Amp and IPTG. This assay was performed with three biological replicates each time and was repeated more than three times. After incubation at 37°C for 12–16 hours, photographs were taken to document the results.

### Pulldown assay for protein‒protein interactions

The pMAL c5X plasmid harboring the toxin genes (MBP-toxin) and the pACYC Duet-1 plasmid containing the immunity gene (immunity-His_6_) were simultaneously transformed into *E. coli* BL21 (DE3) cells. The proteins were subsequently expressed and purified. The protein mixture was then incubated with Ni^2+^ beads (GE Healthcare) for 2 hours and washed with buffer (200 mM imidazole, 20 mM Tris HCl [pH 7.4], 200 mM NaCl and 5% glycerol). The bound proteins were retained on Ni^2+^ beads, and the unbound proteins were eluted. Additionally, bound or unbound proteins on the amylose resin (New England Biolabs) were analyzed via a similar approach. The amylose resin-bound samples were then subjected to SDS-PAGE, and three biological replicates were performed.

### *In vitro* nuclease activity of the toxin protein

A pMAL-c5X plasmid containing the toxin gene with a truncated N-terminus was constructed. The toxin protein fused to the MBP tag was subsequently expressed. The immunity protein was expressed with a His_6_-tag fusion using the pACYC Duet-1 plasmid. The proteins were overexpressed and purified using Ni^2+^ beads or amylose resin and used for the nuclease activity test. The DNA substrate for the *in vitro* assay was the genomic DNA of DK1622. Genomic DNA was extracted using a Tiangen Bacterial DNA Kit (Tiangen, China). Subsequently, 10 mg/mL purified toxin protein and 2 µg of genomic DNA or plasmid were incubated for 40 minutes at 37°C in reaction buffer containing 20 mM Tris HCl (pH 7.4), 200 mM NaCl, and 5% glycerol. Digestion was performed by the known nuclease DNase I used as a positive control. Both incubation with the immunity protein and incubation with a mixture of the immunity protein and DNase I were performed. The reactions were repeated more than three times. The digestion of the DNA mixtures was stopped by the addition of an equal volume of phenol-chloroform isoamyl alcohol, and the extracted DNA mixtures were examined via 0.8% agarose gel electrophoresis. The gels were stained with ethidium bromide and then photographed.

### Sequence homology analysis

We used NCBI BLAST (https://blast.ncbi.nlm.nih.gov/Blast.cgi) to compare the amino acid sequences of homologous proteins of toxins and immunity proteins, and the comparison results were visualized and edited using Jalview software. Weblogo (https://weblogo.berkeley.edu/logo.cgi) was used to generate a schematic diagram of the amino acid sequence alignment.

### Prediction and analysis of toxin immunity complex structures

AlphaFold2 (51) was used on an online server to predict the structure of a protein on the basis of its sequence, which involves placing the protein sequences and other optional prediction parameters, submitting the job, and waiting for the prediction to be completed. Once the prediction was completed, the predicted protein structure file and other output files were downloaded, or the results were viewed directly on the website. The three-dimensional structure of the protein was analyzed and displayed using PyMoL. Modeling of protein and DNA complexes was done with NPDock (https://genesilico.pl/NPDock/). Analysis of protein complex interactions and binding free energy was conducted using PDBePISA (https://www.ebi.ac.uk/pdbe/pisa/).

### Comparison of protein complex structures

The protein complex results obtained from AlphaFold modeling were saved as PDB files. The required protein structure PDB files were uploaded to the online TM-align. We assessed the structural similarity between each pair of toxin–immunity protein complex models using TM alignment (52). The TM-scores of the different complexes were compared to evaluate their structural similarity.

### Analysis of the diversity of the toxin–immunity protein system in *Myxococcus*

We used BLASTn with the four toxin–immunity protein systems from *M. xanthus* as queries to search the complete genomes of *Myxococcus* species, applying an E value threshold of less than 1e−50. On this basis, we have identified toxin–immunity protein pairs, along with their adjacent genes.

## SUPPLEMENTAL MATERIAL

Data set S1 mbio.03902-24-s0001.xlsx Toxin gene transcript levels in the wild-type DK1622 strain and the immunity-deficient mutant strain.

Data set S2 mbio.03902-24-s0002.xlsx Information on four toxin-immunity protein gene clusters across complete *Myxococcus* genomes.

Supplemental Material mbio.03902-24-s0003.pdf Fig. S1 to S11, Tables S1 to S3, and captions for Data sets S1 and S2.

ASM does not own the copyrights to Supplemental Material that may be linked to, or accessed through, an article. The authors have granted ASM a non-exclusive, world-wide license to publish the Supplemental Material files. Please contact the corresponding author directly for reuse.
