# Supplementary material for: Differential crosstalk between toxin-immunity protein homologs divides Myxococcus nonself siblings into close and distant social relatives
Source: mBio. 2025 Mar 28;16(5):e03902-24. doi: 10.1128/mbio.03902-24 (PMC12077131; doi:10.1128/mbio.03902-24)
Supplement: Supplemental Material — Fig. S1 to S11, Tables S1 to S3, and captions for Data sets S1 and S2. [file mbio.03902-24-s0003.pdf]

Supplementary Materials for

Differential crosstalk between toxin-immunity protein homologs divides *Myxococcus* nonself-siblings into close and distant social relatives

Feng Wang[1], Jing Luo[1], Zheng Zhang[1], Ya Liu[1,2], Duo hong Sheng[1], Li Zhuo[1], Yue-zhong Li[1]*

*Corresponding author. Email: lilab@sdu.edu.cn (Yue-zhong Li); ORCID ID: 0000-0001-8336-6638.

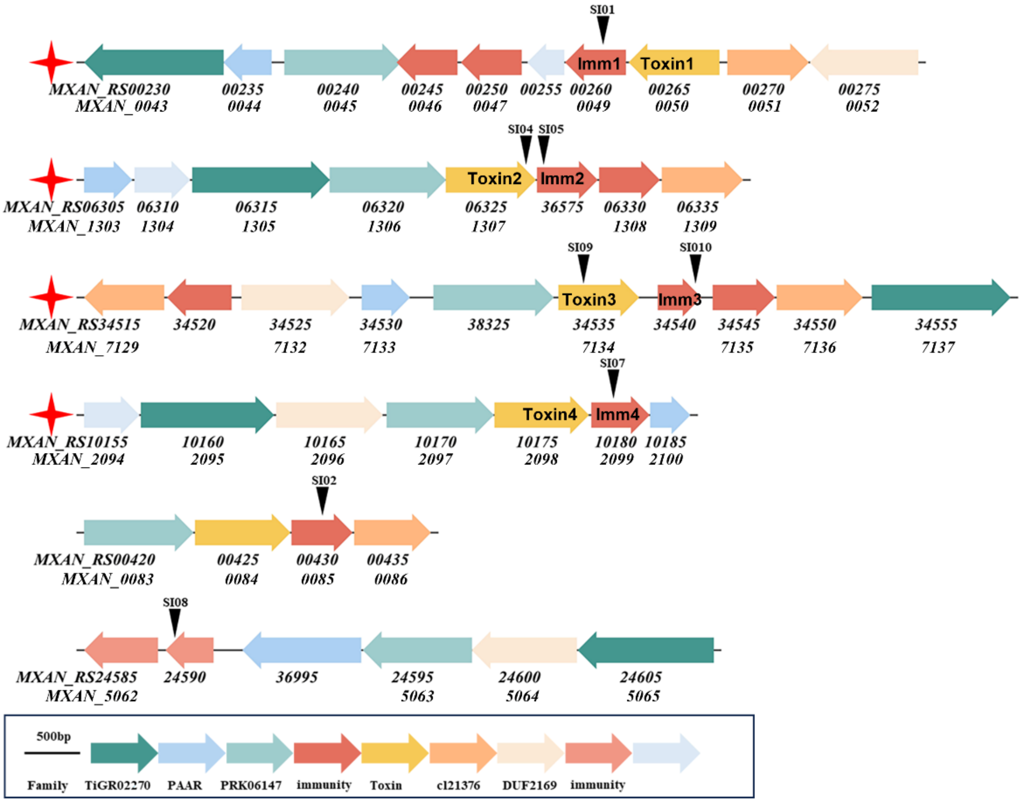

Supplementary Figure S1. The six homologous genetic loci of toxin-immunity protein systems in the *M. xanthus* DK1622 genome, revealed from the random insertion screening using the pMiniHimar-lacZ plasmid. Different functional proteins are marked with different colors. The black triangle indicates the insertion position. The numbers below each gene cluster represent the corresponding gene numbers. Asterisks mark the four recognition-related gene clusters that are the focus of this study. The studied four toxin and immunity gene pairs are highlighted with their names in the diagrammatic boxes.

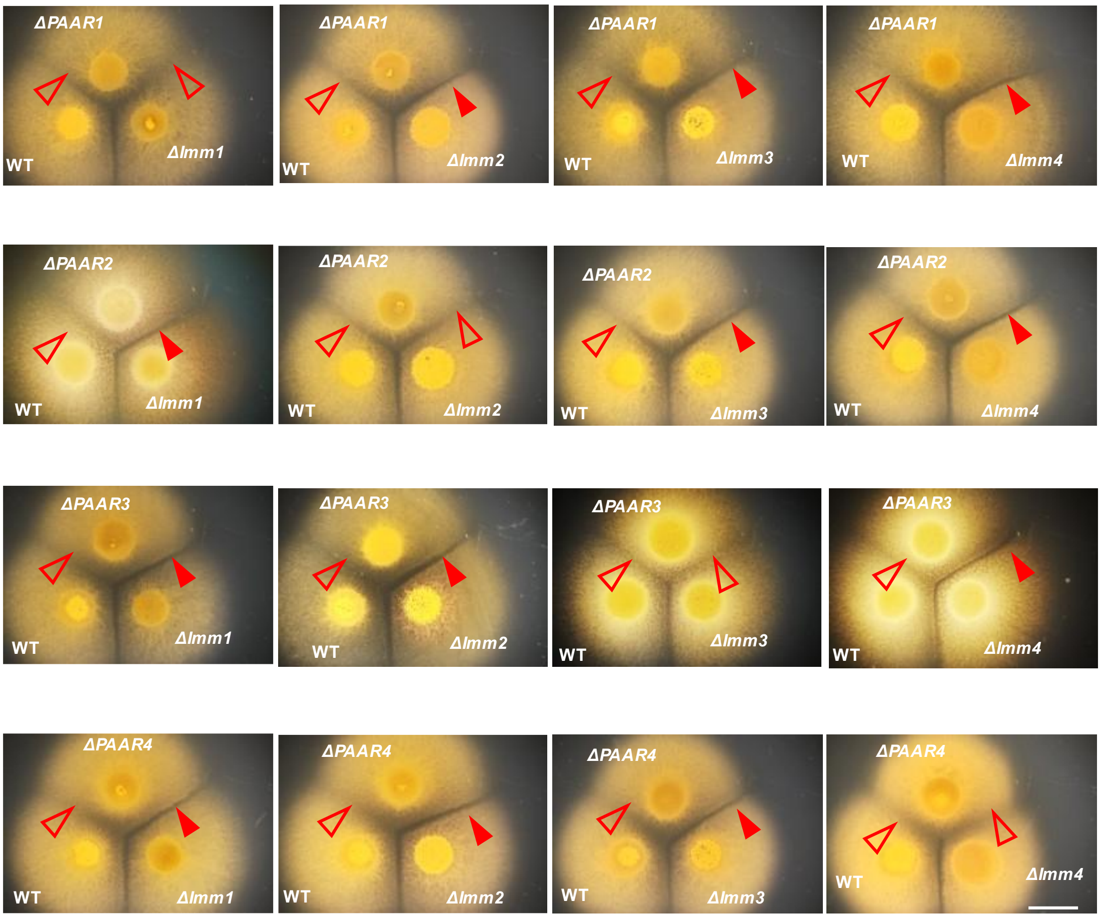

Supplementary Figure S2. The colony boundary formation of the *PAAR1-4* strains with the wild type and corresponding *Imm1-4* strains. The solid triangle indicates where a boundary exists, and the hollow triangle indicates the area where no boundary is observed. The scale bar represents 5 mm.

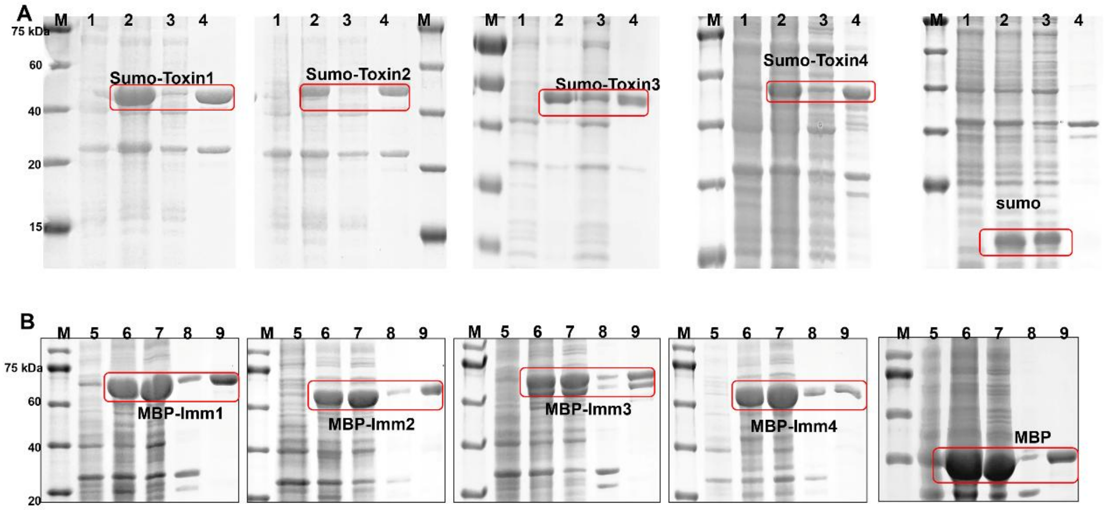

Supplementary Figure S3. Expression and purification of toxin and immunity proteins. (A) Expression of SUMO-tagged toxin protein. M: molecular marker; lanes 1-4 correspond to: 1, whole cell lysate without IPTG-induction; 2, whole cell lysate with IPTG-induction; 3, supernatant following cell lysis; 4, pellet after cell lysis (inclusion bodies). (B) Expression and purification of MBP-tagged immunity protein. M: molecular marker; lanes 5-9 correspond to: 5, whole cell lysate without IPTG-induction; 6, whole cell lysate with IPTG induction; 7, supernatant following cell lysis; 8, wash buffer fraction; 9, elution buffer fraction.

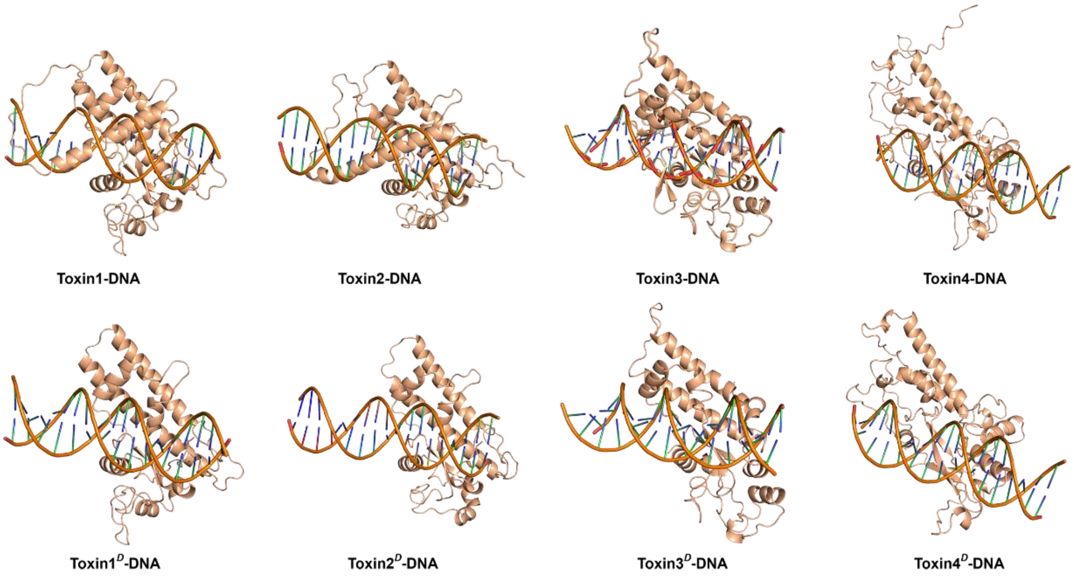

Supplementary Figure S4. The complex structure of complete and truncated toxin proteins bound to double-stranded DNA, modelled using the online program NPDock (https://genesilico.pl/NPDock/) with the default parameters.

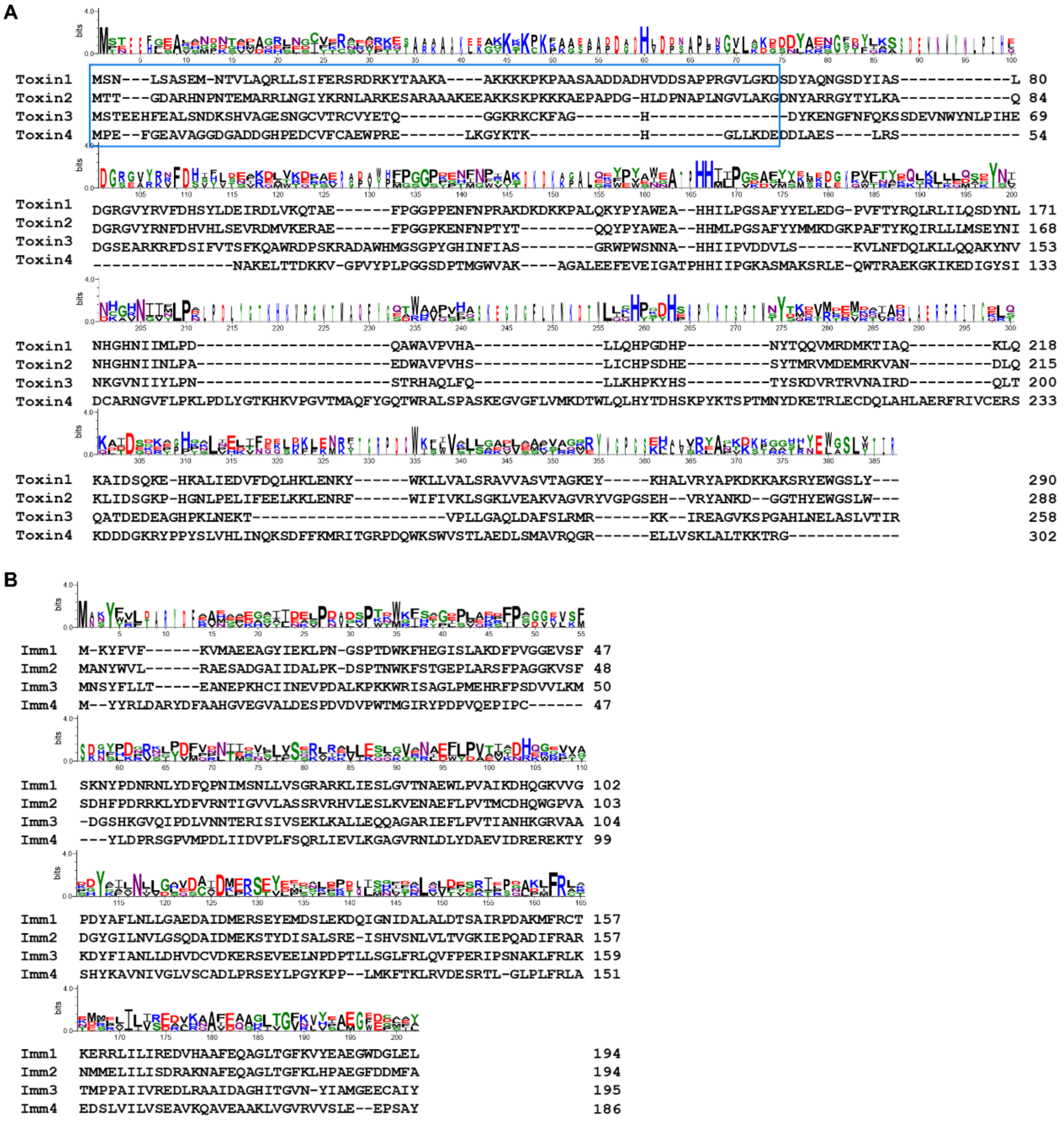

Supplementary Figure S5. Multiple sequence alignment of the four toxin (A) and immunity (B) proteins. The N-terminal region of toxin is marked in blue frame.

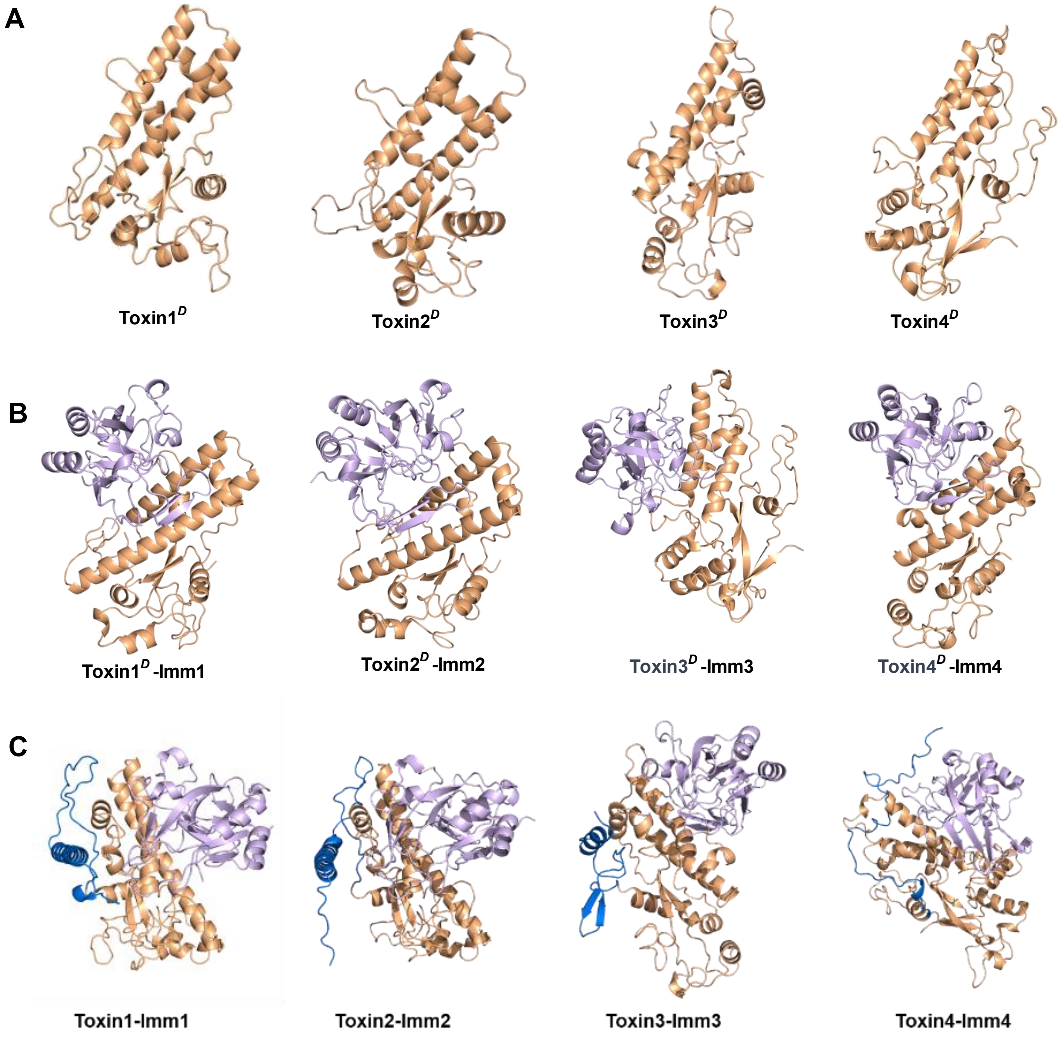

Supplementary Figure S6. Structural modeling of the toxin and toxin-immunity protein complexes using AlphaFold2. (A) Modeled structure of toxin protein with the N-terminal region removed. (B) Complex structure of truncated toxin protein with the removal of N-terminal region bound to its corresponding immunity protein. (C) Complex structure of the full-length toxin protein bound to its corresponding immunity protein.

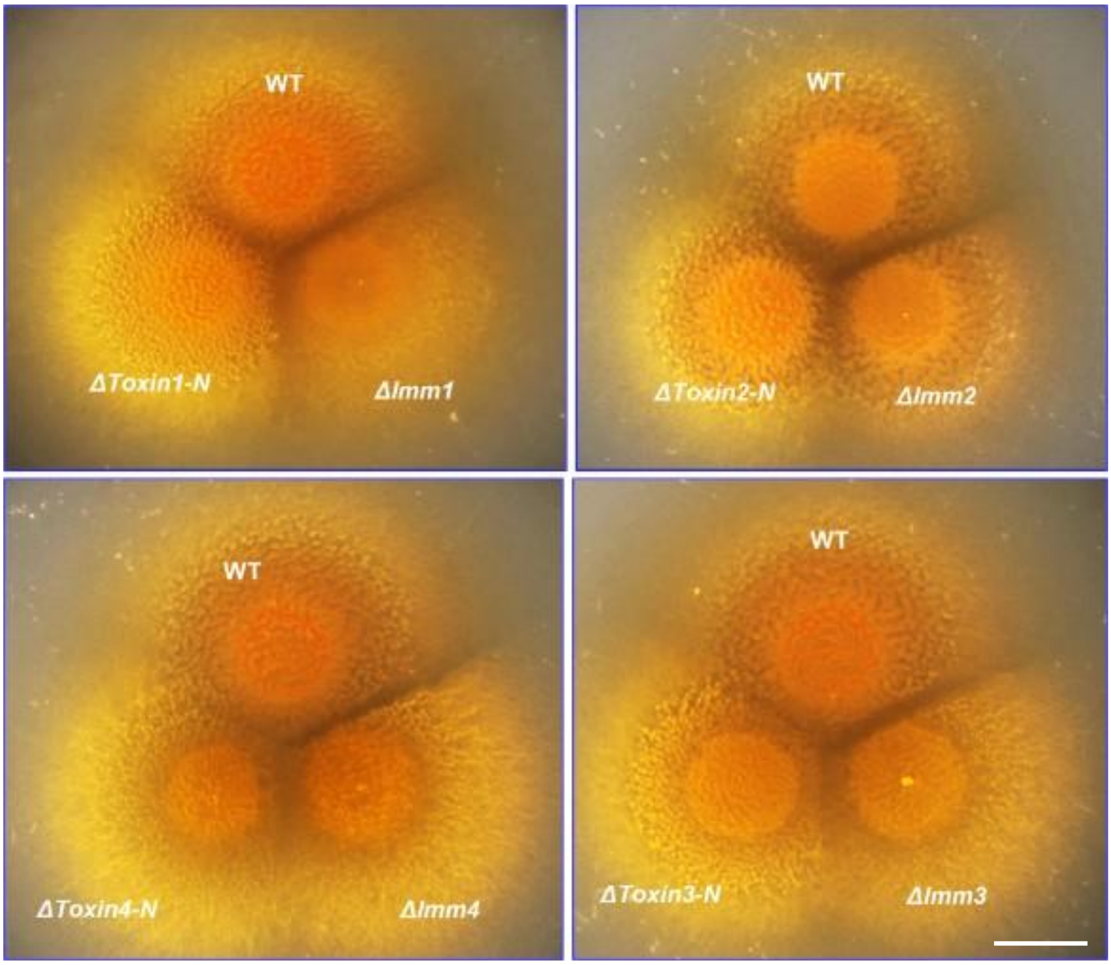

Supplementary Figure S7. Colony boundary formation assay between the strains with the toxin genes truncated of N-terminal region and the wild type strain DK1622, as well as the mutants with the deletion of their corresponding immunity genes, on CTT agar. The scale bar represents 5 mm.

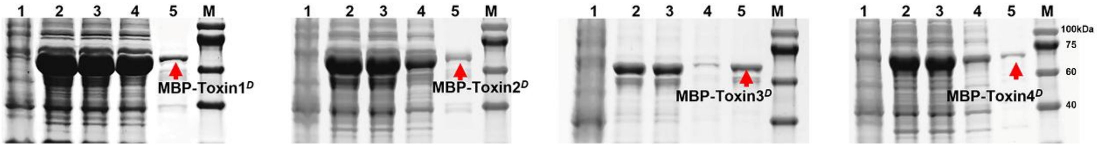

Supplementary Figure S8. The expression and purification of truncated toxin proteins tagged with an MBP. M: Marker, Lanes 1-5 represent: (1) whole cell proteins without the IPTG-induction, (2) whole cell proteins with the IPTG-induction, (3) supernatant following cell disruption, (4) wash buffer eluate, (5) elution buffer eluate.

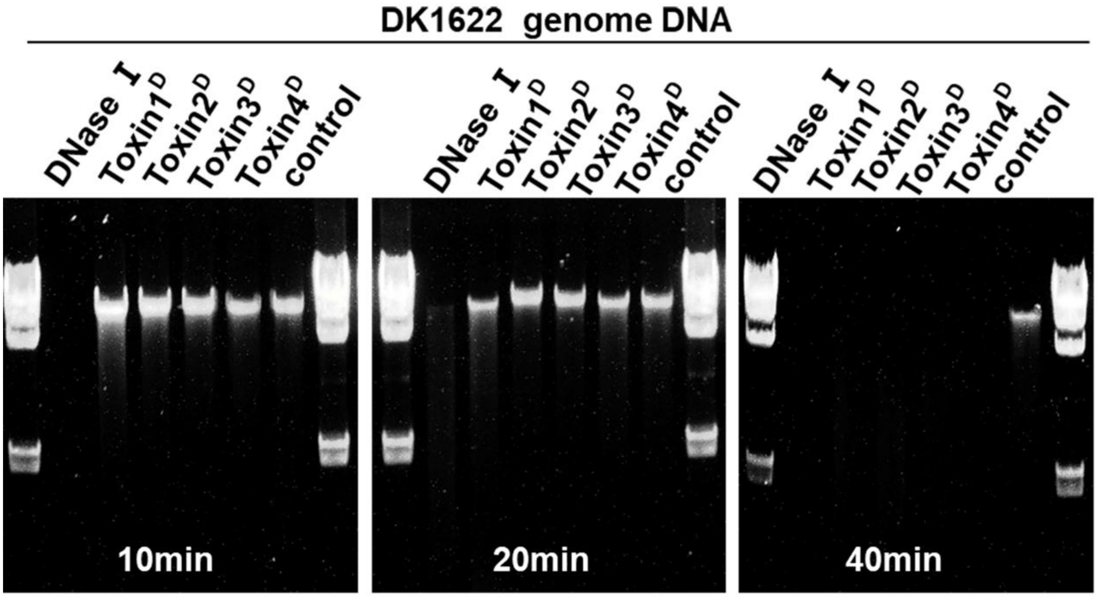

Supplementary Figure S9. In vitro nuclease activity assay of toxin proteins. DNase I was used as a positive control, the *E. coli* extract with the plasmid expressing the MBP-tag only was added to the negative control, and the λ-HindIII-digested DNA was served as a marker. The reaction products were sampled at 10, 20 and 40 min for detection via agarose gel electrophoresis.

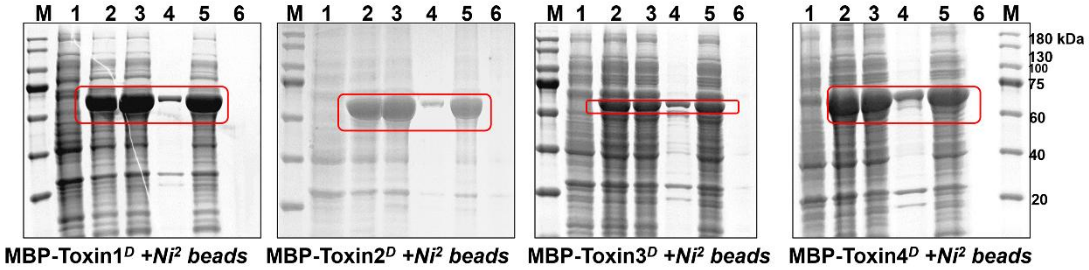

Supplementary Figure S10. Binding assay of MBP-tagged toxin protein to Ni resin, shown with SDS–PAGE. M: Marker; 1: Whole cell lysate without the IPTG-induction; 2: Whole cell lysate with the IPTG-induction; 3: Soluble fraction; 4, pellet after cell lysis (inclusion bodies); 5: Unbound fraction (proteins not bound to Ni resin); 6: Bound fraction (proteins bound to Ni resin).

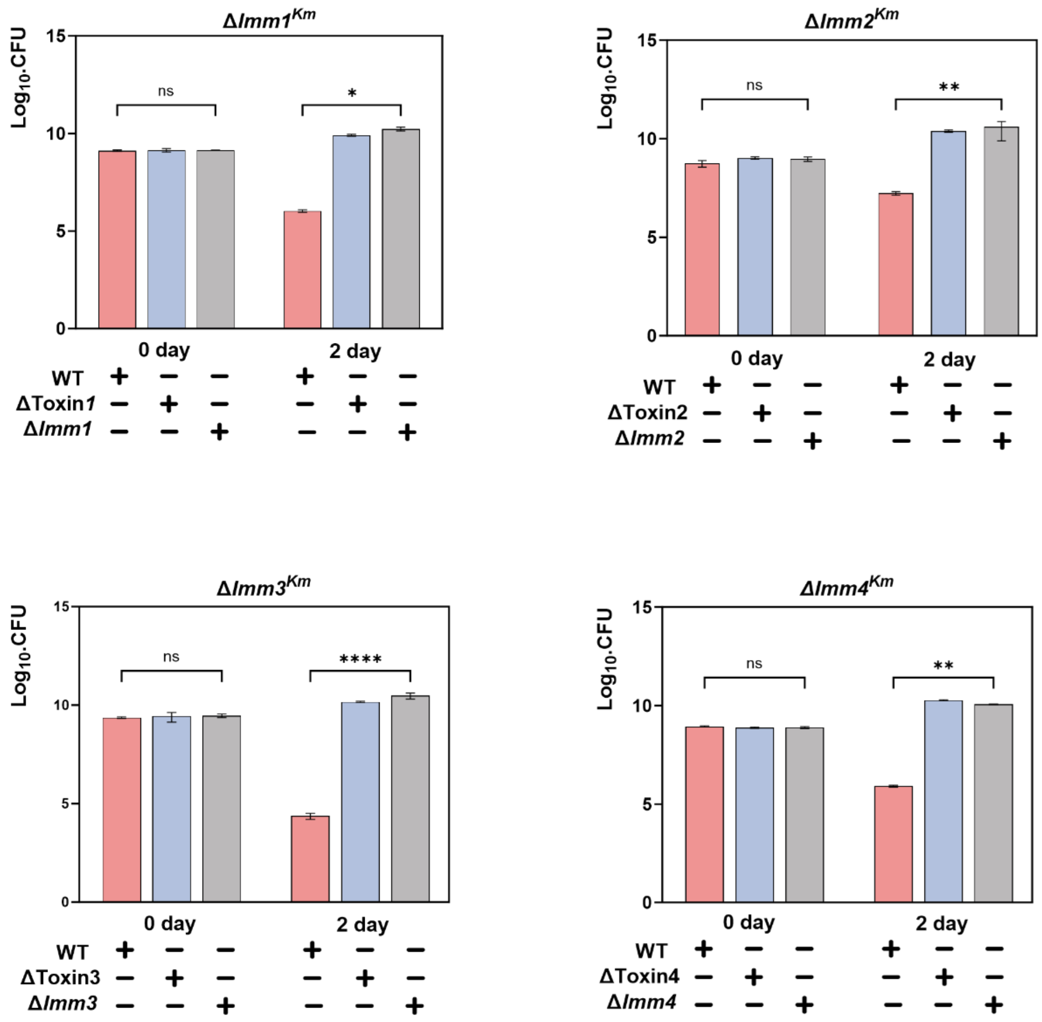

Supplementary Figure S11. Competitive growth assay between kanamycin-resistant Δ*Imm1-4* strains and kanamycin-sensitive wild-type strains, the same Δ*Imm* strains and the corresponding Δ*Toxin* strains in equal cell numbers. The mixture was incubated on CTT medium for 2 days, followed by dilution and plating on kanamycin-containing CTT medium to count CFUs. Three dilutions and three replicates were tested.

Supplementary Table S1. Information of the four toxin (truncated toxin) and immunity proteins.

Locus tag	Name	Length (aa)	MW (kDa)	pI
Immunity protein				
MXAN_RS00260	Imm1	194	21856.79	4.78
MXAN_RS36575	Imm2	194	21491.41	5.35
MXAN_RS34540	Imm3	195	15554.01	5.38
MXAN_RS10180	Imm4	186	20853.05	4.84
Nuclease-toxin				
MXAN_RS00265	Toxin1	290	33407.89	9.19
MXAN_RS06325	Toxin2	288	32948.45	9.26
MXAN_RS34535	Toxin3	258	29360.02	9.22
MXAN_RS10175	Toxin4	302	33902.63	7.65
MXAN_RS00265^{D}	Toxin1^{D}	224	25882.33	8.48
MXAN_RS06325^{D}	Toxin2^{D}	218	25377.81	7.36
MXAN_RS34535^{D}	Toxin3^{D}	214	24547.7	9.52
MXAN_RS10175^{D}	Toxin4^{D}	257	29014.23	9.15

Supplementary Table S2. Strains and plasmids used in this study.

Designation	Genotype or description	Source
**strain**		
M. xanthus		
DK1622	Wild type	D. Kaiser, Stanford University
Δ*Toxin1*	Deletion of *MXAN_RS00265*	This study
Δ*Toxin2*	Deletion of *MXAN_RS06325*	This study
Δ*Toxin3*	Deletion of *MXAN_RS34535*	This study
Δ*Toxin4*	Deletion of *MXAN_RS10175*	This study
Δ*Imm1*	Deletion of *MXAN_RS00260*	This study
Δ*Imm2*	Deletion of *MXAN_RS36575*	This study
Δ*Imm3*	Deletion of *MXAN_RS34540*	This study
Δ*Imm4*	Deletion of *MXAN_RS10180*	This study
Δ*PAAR1*	Deletion of *MXAN_RS00235*	This study
Δ*PAAR2*	Deletion of *MXAN_RS06305*	This study
Δ*PAAR3*	Deletion of *MXAN_RS34530*	This study
Δ*PAAR4*	Deletion of *MXAN_RS10185*	This study
Δ$Imm1^{Km}$	Δ*Imm1* + pSWU19	This study
Δ$Imm2^{Km}$	Δ*Imm2* + pSWU19	This study
Δ$Imm3^{Km}$	Δ*Imm3* + pSWU19	This study
Δ$Imm4^{Km}$	Δ*Imm4* + pSWU19	This study
WT-Tomato	DK1622+ pZJY41+ tdTomato	This study
Δ*Imm1*-Tomato	Δ*Imm1* + pZJY41+ tdTomato	This study
Δ*Imm2*- Tomato	Δ*Imm2*+ pZJY41+ tdTomato	This study
Δ*Imm3*- Tomato	Δ*Imm3*+ pZJY41+ tdTomato	This study
Δ*Imm4*- Tomato	Δ*Imm4*+ pZJY41+ tdTomato	This study
Δ*Imm1*-GFP	Δ*Imm1* + pZJY41+eGFP	This study
E. coli		
DH5α		Life Technologies Inc.
BL21 (DE3)		Life Technologies Inc.
Plasmids		
pBJ113	Gene replacement vector with KG cassette; Kmr	(1)
pZJY41	Stable E. coli–M. xanthus shuttle plasmid; Kmr, Ampr	(2)
pSWU19	Site-specific integration vector with Mx8 attP integration site; Kmr	(3)
pMAL c5X	MBP tag	Biolabs
pACYC Duet-1	His tag	Novagen
pET28a	His tag	Novagen
pET28a- SUMO	His-SUMO tag+ pET28a	This study
Toxin1-pBJ113	*MXAN_RS00265* gene replacement vector with KG cassette; Km^r	This study
Toxin2-pBJ113	*MXAN_RS06325* gene replacement vector with KG	This study

	cassette; Km^r	
Toxin3-pBJ113	*MXAN_RS34540* gene replacement vector with KG cassette; Km^r	This study
Toxin4-pBJ113	*MXAN_RS10175* gene replacement vector with KG cassette; Km^r	This study
Imm1-pBJ113	*MXAN_RS00260* gene replacement vector with KG cassette; Km^r	This study
Imm2-pBJ113	*MXAN_RS36575* gene replacement vector with KG cassette; Km^r	This study
Imm3-pBJ113	*MXAN_RS34545* gene replacement vector with KG cassette; Km^r	This study
Imm4-pBJ113	*MXAN_RS10180* gene replacement vector with KG cassette; Km^r	This study
PAAR1-pBJ113	*MXAN_RS00235* gene replacement vector with KG cassette; Km^r	This study
PAAR2-pBJ113	*MXAN_RS06305* gene replacement vector with KG cassette; Km^r	This study
PAAR3-pBJ113	*MXAN_RS34530* gene replacement vector with KG cassette; Km^r	This study
PAAR4-pBJ113	*MXAN_RS10185* gene replacement vector with KG cassette; Km^r	This study
Toxin1- pMAL c5X	Amp^r, tac promoter, maltose-binding protein fusions, *MXAN_RS00265* insertion in pMAL c5X	This study
Toxin2-pMAL c5X	Amp^r, tac promoter, maltose-binding protein fusions, *MXAN_RS06325* insertion in pMAL c5X	This study
Toxin3- pMAL c5X	Amp^r, tac promoter, maltose-binding protein fusions, *MXAN_RS34540* insertion in pMAL c5X	This study
Toxin4- pMAL c5X	Amp^r, tac promoter, maltose-binding protein fusions, *MXAN_R10175* insertion in pMAL c5X	This study
Imm1- pMAL c5X	Amp^r, tac promoter, maltose-binding protein fusions, *MXAN_RS00260* insertion in pMAL c5X	This study
Imm2- pMAL c5X	Amp^r, tac promoter, maltose-binding protein fusions, *MXAN_RS36575* insertion in pMAL c5X	This study
Imm3- pMAL c5X	Amp^r, tac promoter, maltose-binding protein fusions, *MXAN_RS34545* insertion in pMAL c5X	This study
Imm4- pMAL c5X	Amp^r, tac promoter, maltose-binding protein fusions, *MXAN_R10180* insertion in pMAL c5X	This study
Imm1-pACYC Duet-1	Cm^r, T7 promoter, His tag, *MXAN_RS08760* insertion in pACYC Duet-1	This study
SUMO-Toxin1	*MXAN_RS00265* insertion in pET28a	This study
SUMO-Toxin2	*MXAN_RS06325* insertion in pET28a	This study
SUMO-Toxin3	*MXAN_RS34540* insertion in pET28a	This study
SUMO-Toxin4	*MXAN_RS10175* insertion in pET28a	This study
Imm1-Tomato	*Imm1* + pZJY41+ tdTomato	This study

Imm2- Tomato	*Imm2*+ pZJY41+ tdTomato	This study
Imm3- Tomato	*Imm3*+ pZJY41+ tdTomato	This study
Imm4- Tomato	*Imm4*+ pZJY41+ tdTomato	This study
Imm1-GFP	*Imm1* + pZJY41+eGFP	This study

Supplementary Table S3. Primers used in this study.

Primer	Sequence (5′-3′)
Imm1-L-up	TGAAACCTGTCAGTCCC
Imm1-L-down	TCATTCGAGATTTTTCCCCATCATGTTCGAAG
Imm1-R-up	CTTCGAACATGATGGGGAAAAATCTCGAATGA
Imm1-R-down	GTTCAGGCACCATACAA
Imm2-L-up	GTATCCCTCCATCGGCTTTG
Imm2-L-down	AACCCAATAGTCCATCACCTACCAAAGGCTGCCCCA
Imm2-R-up	TGGGGCAGCCTTTGGTAGGTGATGGACTATTGGGTT
Imm2-R-down	CAAGGACCGAGCGAAACG
Imm3-L-up	TTCTCCTGGAACGGATTGA
Imm3-L-down	TAGGAGTGGCATACGGTGTTCATCTACCGGATGGTCACGAGA
Imm3-R-up	TCTCGTGACCATCCGGTAGATGAACACCGTATGCCACTCCTA
Imm3-R-down	AGGAGCGGCATTCATTTT
Imm4-L-up	CGAGCTCCTGGGATTGCGGAAGGGATA
Imm4-L-down	ACACCACCCGGACTCCCACGCACGGGATGGGCTCTTG
Imm4-R-up	CAAGAGCCCATCCCGTGCGTGGGAGTCCGGGTGGTGT
Imm4-R-down	GCTCTAGAGAGGAGCCCTTCCCTGTTT
Toxin1-L-up	CCTTTTCATCAGACAG
Toxin1-L-down	ACCATACAACGTGAGAGTAATTGAACCAGTCAT
Toxin1-R-up	ATGACTGGTTCAATTACTCTCACGTTGTATGGT
Toxin1-R-down	GACTGACGGGGTTATCG
Toxin2-L-up	GGAATTCTTTGGGACAGGAGTCGTTGCTC
Toxin2-L-down	TACCAAAGGCTGCCCCACTCATAGTGCCGGGCGTCACCGGTTGTC
Toxin2-R-up	GACAACCGGTGACGCCCGGCACTATGAGTGGGGCAGCCTTTGGTA
Toxin2-R-down	CCCAAGCTTCCTCCAGCACCTGTTTCA
Toxin3-L-up	CGAGCTCGAGGCAGTATCGCTAAAGGG
Toxin3-L-down	TGAGTTCATGGACGGCACCTCTTCAGTGCTCATGAC
Toxin3-R-up	GTCATGAGCACTGAAGAGGTGCCGTCCATGAACTCA
Toxin3-R-down	CGGGATCCAATCGTCCTGGATGTTTCTC
Toxin4-L-up	GGAATTCCGCTTCCAGTTGCTGTCATTG
Toxin4-L-down	ACACCTCCGCGACAATTGAGGCTCAAGCCTCCGGATCT
Toxin4-R-up	AGATCCGGAGGCTTGAGCCTCAATTGTCGCGGAGGTGT
Toxin4-R-down	CCCAAGCTTGGACGGTTACGCAGAGCAT
PAAR1-L-up	GGAATTCCCACTGCCGGGACTCCATCT
PAAR1-L-down	GCATGAGCGCGACCGTTGGCCGTCTGTGAAAAGGAGCT
PAAR1-R-up	AGCTCCTTTTCACAGACGGCCAACGGTCGCGCTCATGC
PAAR1-R-down	CCCAAGCTTGCCTCACCGGCTTCAAGGTC
pBJ113-up	GGGGATCCTCTAGAGTCGACCT
pBJ113-down	GGGTACCGAGCTCGAATTCA
28a-up	GAGCTCGAATTCGGATCCG

Primer	Sequence (5′-3′)
28a-down	CGTCGACAAGCTTGCGGC
Tox1N-up	cggccgcaagcttgtcgacgGTCTTTGCCGAGCACGCC
Tox1N-down	gcggatccgaattcgagctcATGAGCAACCTGAGTGCGAGC
Tox1D-up	cggccgcaagcttgtcgacgTTAATAGAGAGAGCCCCACTCATAAC
Tox1D-down	gcggatccgaattcgagctcAGCGACTACGCGCAGAACG
pMALc5x-cx-F	GGTCGTCAGACTGTCGATGAAGCC
pMALc5x-cx-R	TGTCCTACTCAGGAGAGCGTTCAC
T1D-MBP-up	AGGGAAGGATTTCACATATGAGCGACTACGCGCAGAACG
T1D -MBP-down	CCTGCAGGGAATTCGGATCCTTAATAGAGAGAGCCCCACTCATAAC
Toxin1-NR-up	TGAATTCGAGCTCGGTACCCCATTCGGCATTGGTGACACC
Toxin1-NR-down	TGGGTACGCGCAGCGACTACGCGCAGAACG
Toxin1-NL-up	GTAGTCGCTGCGCGTACCCACCTCTCAC
Toxin1-NL-down	GTCGACTCTAGAGGATCCCCGCGCGGAGACGCTGGAGC
Toxin2-NR-up	TGAATTCGAGCTCGGTACCCGGAAGTGGTCCGAGAACGACA
Toxin2-NR-down	GAGCTCTGCCGATAACTATGCCCGCCGCG
Toxin2-NL-up	CATAGTTATCGGCAGAGCTCCTCTGGACG
Toxin2-NL-down	GTCGACTCTAGAGGATCCCCGGAGCGAGGCCTTGACCTG
Toxin3-NR-up	TGAATTCGAGCTCGGTACCCCTGTTCTAGCAGGGCCTTGAGC
Toxin3-NR-down	TGAGACAGTCGACTACAAGGAGAATGGATTCAACTTC
Toxin3-NL-up	CCTTGTAGTCGACTGTCTCACATGCAGTGACGTC
Toxin3-NL-down	GTCGACTCTAGAGGATCCCCGTGGCGTACTCAGGACTGACTCG
Toxin4-NR-up	AGGTCGTCGGCTCAAGCCTCCGGATC
Toxin4-NR-down	GTCGACTCTAGAGGATCCCCCTGCCGGCTCCGGATCTG
Toxin4-NL-up	TGAATTCGAGCTCGGTACCCTAATACACACACACCTCCGCGA
Toxin4-NL-down	AAGATCCGGAGGCTTGAGCCGACGACCTGGCGGAGAGC
Imm1-up	CACATATGTCCATGGGCGGCGTGAAATATTTTGTCTTCAAGGTCATG
Imm1-down	GATCCGTCGACGATATCGCGTCAGAGCTCCAGTCCGTCCC
Imm2-up	AGGGAAGGATTTCACATATGATGGCTAATTACTGGGTGTTGCG
Imm2-down	CCTGCAGGGAATTCGGATCCTCAAGCGAACATGTCGTCGA
Imm3-up	AGGGAAGGATTTCACATATGATGAACTCATACTTCCTCCTCACCG
Imm3-down	CCTGCAGGGAATTCGGATCCTCAATAGATAGCGCATTCTTCGC
Imm4-up	AGGGAAGGATTTCACATATGGTGTATTACAGGTTGGATGCGAGG
Imm4-down	CCTGCAGGGAATTCGGATCCTCAGTATGCGGACGGCTCTT
Tox1sumo-up	gatccgaattcgagctccgtATGAGCAACCTGAGTGCGAGC
Tox1sumo-down	gtgcggccgcaagcttgtcgTTAATAGAGAGAGCCCCACTCATAAC
Tox2sumo-up	gatccgaattcgagctccgtATGACAACCGGTGACGCCC
Tox2sumo-down	gtgcggccgcaagcttgtcgCTACCAAAGGCTGCCCCAC
Tox3sumo-up	gatccgaattcgagctccgtATGAGCACTGAAGAGCATTTCGA
Tox3sumo-down	gtgcggccgcaagcttgtcgCTACCGGATGGTCACGAGACTT
Tox4sumo-up	gatccgaattcgagctccgtATGCCTGAGTTTGGCGAAGC
Tox4sumo-down	gtgcggccgcaagcttgtcgCTAACCGCGCGTCTTCTTCG

Primer	Sequence (5′-3′)
Tox-Imm1-up	AGGGAAGGATTTCACATATGATGAGCAACCTGAGTGCGAGC
Tox-Imm1-down	CCTGCAGGGAATTCGGATCCTCAGAGCTCCAGTCCGTCCC
Tox-Imm2-up	AGGGAAGGATTTCACATATGATGACAACCGGTGACGCCC
Tox-Imm2-down	CCTGCAGGGAATTCGGATCCTCAAGCGAACATGTCGTCGA
Tox-Imm3-up	AGGGAAGGATTTCACATATGATGAGCACTGAAGAGCATTTCGA
Tox-Imm3-down	CCTGCAGGGAATTCGGATCCTCAATAGATAGCGCATTCTTCGC
Tox-Imm4-up	AGGGAAGGATTTCACATATGATGCCTGAGTTTGGCGAAGC
Tox-Imm4-down	AGGGAAGGATTTCACATATGATGCCTGAGTTTGGCGAAGC
T2D-MBP-up	AGGGAAGGATTTCACATATGGATAACTATGCCCGCCGCG
T2D -MBP-down	CCTGCAGGGAATTCGGATCCCTACCAAAGGCTGCCCCAC
T3D-MBP-up	AGGGAAGGATTTCACATATGGACTACAAGGAGAATGGATTCAACTTC
T3D -MBP-down	CCTGCAGGGAATTCGGATCCCTACCGGATGGTCACGAGACTT
T4D-MBP-up	AGGGAAGGATTTCACATATGGACGACCTGGCGGAGAGC
T4D -MBP-down	CCTGCAGGGAATTCGGATCCCTAACCGCGCGTCTTCTTCG
Mbp-BamHI-Up	GGATCCGAATTCCCTGCAG
Mbp-BamHI-down	CATATGTGAAATCCTTCCCTCGA

1. Julien B, Kaiser AD, & Garza A (2000) Spatial control of cell differentiation in Myxococcus xanthus. *Proc Natl Acad Sci U S A* 97(16):9098-9103.
2. Zhao JY*, et al.* (2008) Discovery of the autonomously replicating plasmid pMF1 from Myxococcus fulvus and development of a gene cloning system in Myxococcus xanthus. *Appl Environ Microbiol* 74(7):1980-1987.
3. Wu SS & Kaiser D (1995) Genetic and functional evidence that Type IV pili are required for social gliding motility in Myxococcus xanthus. *Mol Microbiol* 18(3):547-558.

Supplementary Data

Supplementary Data S1 Toxin gene transcript levels in the wild-type DK1622 strain and the immunity-deficient mutant strain.

Supplementary Data S2 Information on four toxin-immunity protein gene clusters across complete Myxococcus genomes.
